# An Energy Conserving and Transmission Radius Adaptive Scheme to Optimize Performance of Energy Harvesting Sensor Networks

**DOI:** 10.3390/s18092885

**Published:** 2018-08-31

**Authors:** Xin Ju, Wei Liu, Chengyuan Zhang, Anfeng Liu, Tian Wang, Neal N. Xiong, Zhiping Cai

**Affiliations:** 1 School of Information Science and Engineering, Central South University, Changsha 410083, China; XinjuTry@csu.edu.cn (X.J.); afengliu@mail.csu.edu.cn (A.L.); 2 School of Informatics, Hunan University of Chinese Medicine, Changsha 410208, China; weiliu@csu.edu.cn; 3 The State Key Laboratory of Industrial Control Technology, Zhejiang University, Hangzhou 310027, China; 4 College of Computer Science and Technology, Huaqiao University, Xiamen 361021, China; wangtian@hqu.edu.cn; 5 Department of Mathematics and Computer Science, Northeastern State University, Tahlequah, OK 74464, USA; xiongnaixue@gmail.com; 6 Department of Network Engineering, School of Computer, National University of Defense Technology, Changsha 410073, China; zpcai@nudt.edu.cn

**Keywords:** energy harvesting wireless sensor networks, energy conserving, transmission radius adaptive, delay, cost

## Abstract

In energy harvesting wireless sensor networks (EHWSNs), the energy tension of the network can be relieved by obtaining the energy from the surrounding environment, but the cost on hardware cannot be ignored. Therefore, how to minimize the cost of energy harvesting hardware to reduce the network deployment cost, and further optimize the network performance, is still a challenging issue in EHWSNs. In this paper, an energy conserving and transmission radius adaptive (ECTRA) scheme is proposed to reduce the cost and optimize the performance of solar-based EHWSNs. There are two main innovations of the ECTRA scheme. Firstly, an energy conserving approach is proposed to conserve energy and avoid outage for the nodes in hotspots, which are the bottleneck of the whole network. The novelty of this scheme is adaptively rotating the transmission radius. In this way, the nodes with maximum energy consumption are rotated, balancing energy consumption between nodes and reducing the maximum energy consumption in the network. Therefore, the battery storage capacity of nodes and the cost on hardware. Secondly, the ECTRA scheme selects a larger transmission radius for rotation when the node can absorb enough energy from the surroundings. The advantages of using this method are: (a) reducing the energy consumption of nodes in near-sink areas, thereby reducing the maximum energy consumption and allowing the node of the hotspot area to conserve energy, in order to prevent the node from outage. Hence, the network deployment costs can be further reduced; (b) reducing the network delay. When a larger transmission radius is used to transmit data in the network, fewer hops are needed by data packet to the sink. After the theoretical analyses, the results show the following advantages compared with traditional method. Firstly, the ECTRA scheme can effectively reduce deployment costs by 29.58% without effecting the network performance as shown in experiment analysis; Secondly, the ECTRA scheme can effectively reduce network data transmission delay by 44–71%; Thirdly, the ECTRA scheme shows a better balance in energy consumption and the maximum energy consumption is reduced by 27.89%; And lastly, the energy utilization rate is effectively improved by 30.09–55.48%.

## 1. Introduction

The internet of things (IoT) [1,2,3] is becoming a pervasive paradigm and will have a significant impact on future applications in many fields, including environmental monitoring [4,5,6], industrial manufacture [7,8,9], telecommunications [10,11,12], intelligent transportation [13,14,15], e-health [15,16,17], and social networks [17,18,19]. IoT composed of numerous ubiquitous sensor-based devices can enrich data awareness and acquisition [20,21], combined with current fast-growing computational intelligent technologies [22,23] to make intelligent decisions [24,25], and thus, form smart IoT, which obtain extensive attention from academic and industrial researchers [26,27]. Nowadays, smart sensors based on IoT are playing a more and more fundamental role in many applications [28,29]. For example, they play a key role in long-term monitoring of a wide range of ecological environments [30], real-time monitoring and intelligent decision-making on traffic status [31], and are also widely used in agriculture, military, industrial automation, and many other applications [32,33,34,35]. However, there are several challenges related to the mass promotion for smart IoT applications, and some of these challenges are to make IoT work with low cost, as well as a green and an energy-efficient paradigm [36,37,38]. In the beginning, the sensor node is small in size and is powered by battery, so it requires a fine plan for energy use to extend its life as much as possible. With the development of microelectronics manufacture polytechnics, energy harvesting wireless sensor networks (EHWSNs) [39,40]—whose nodes can charge energy themselves by harvesting the energy of the surrounding environment—also develop. In EHWSNs, sensor nodes equipped with equipment that absorbs energy from the surrounding environment can replenish energy from the surrounding environment, so that they can support long-term work in an unmanned environment that does not require an energy supply, making EHWSNs more widely applicable than the usual wireless sensor networks (WSNs) [40,41]. For example, for an EHWSN based on solar energy, its sensor nodes add solar panels that can absorb solar energy, in order to absorb solar energy in time and to charge nodes. [42,43]. At the same time, some sensor devices with a wind wheel can replenish energy through wind energy, while other sensor devices can replenish electric energy by heat energy and vibrational energy [44]. Since these sensor nodes can replenish electric energy from the surrounding environment and achieve long-term monitoring of areas requiring inspection, theoretically, after deployment, it also can be called a green network, green computing, or green IoT [45,46,47,48]. Compared with WSNs, EHWSNs have a significant advantage in application scenarios, performance properties, and development prospects, thus, they are getting focused attention from researchers [49,50].

However, in EHWSNs, there are three challenging issues worth studying: The first is reducing costs for EHWSNs. The second concerns energy-efficiency or green computing. Finally, the third involves network performance optimization, especially in reducing network delays.

(1) First, the issue of how to reduce the cost of network deployment. The sensor node of EHWSNs need to be equipped with energy harvesting hardware to absorb energy from the surrounding environment, which will increase construction cost. For example, the solar collector of solar energy harvesting wireless sensor networks mainly consist of a power controller and solar panel, in which the solar panel is used to absorb solar energy. Obviously, the larger the solar panel, the more energy is absorbed per unit time, but the larger the solar panel, the greater the construction cost. Accordingly, the construction cost of the power controller is also increased. Therefore, from the perspective of economic cost, the deployment cost of the network should be as low as possible. Especially for EHWSNs, the number of sensor nodes is huge, so the small cost increase of each node will have a huge impact on the total network deployment cost. Based on this, although EHWSNs can absorb energy from a surrounding environment, cost-effective use of energy is still an important issue worth studying. In EHWSNs, the energy issue involves not only how to effectively use it to improve network performance, but also how to reduce the hardware cost of the sensor node and build the network with minimum deployment cost.
(2) The issue for energy-efficiency in EHWSNs. The principle of energy use in EHWSNs is very different from traditional sensor networks. In traditional sensor networks that cannot replenish energy from the surrounding environment, the principle of its energy use is to minimize energy consumption and extend network lifetime. Instead, in an energy harvesting network, the principle of its energy use is to maximize energy consumption, which is called the principle of energy neutral [39,40,41,42,43,44]. For example, in solar energy harvesting wireless sensor networks, instead of reducing energy consumption, the key is how to utilize the absorbed energy as much as possible to improve network performance during the daytime, especially at noon when solar energy is sufficient. The node cannot conserve all the energy absorbed from solar energy when the battery is fully charged, for the battery volume is limited. Therefore, it is an effective way to utilize solar energy as much as possible [39,43].

An important feature of energy consumption in wireless sensor networks is uneven energy consumption [51,52], because its data transmission is centered on the sink, which shows a many-to-one data collection feature. Therefore, the energy consumption around the sink is much higher than that of other regions in hotspots, and the energy consumption of the far sink region node is much smaller than that of hotspots, which causes premature death of hotspot nodes in ordinary WSNs, due to high energy consumption, thus forming a so-called “energy hole” and making the network die prematurely [11,30,36]. For EHWSNs, although energy can be absorbed to reduce energy stress, the imbalance of network energy consumption still has the following two effects on the network: (a) Since the energy consumption of nodes in a hotspot region is much higher than that in other regions, when the energy absorbed by the environment is insufficient to support energy consumption itself, the node will stop operating data to protect themselves (which is called outage of the nodes). For example, in a solar sensor network, when the solar panel is fixed, if the absorbed solar energy is too little to fully charge the battery during a continuously cloudy day, then the energy of the hotspot region nodes may be exhausted [39,40]. (b) Although increasing the solar panel to make the nodes absorb enough solar energy on a continuously cloudy day can support large energy consumption, and ensure the sensor node does not undergo outage in order to maintain the normal network running [43,44], in a homogeneous network, the hotspot area is a relatively small area for the entire network. In other words, when the energy absorbed by the solar collector can meet the energy consumption of the high energy consumption hotspot nodes, the energy of the majority of non-hotspot nodes must have a large surplus. That is to say, the energy is not fully utilized. Also, according to related research, there is up to 90% of energy remaining in the whole network in such cases [30,36,51]. What is worse, the solar collector is designed to meet the maximum energy consumption requirement, but in fact, the solar collector of most nodes does not need to be so large, which also increases the network deployment cost greatly, and is economically uneconomical.

(3) The issue of reducing delay. Data is routed from the source node to the sink via multi-hop routing, and one of the important performance parameters is delay [7,9,15,28,30,31,52], which generally refers to the time that the data is generated from the source node until the sink successfully receives the data packet. Obviously, the smaller the delay, the better, because in some delay-sensitive applications, it makes sense to route data to the sink quickly because delayed data transmission may cause disastrous consequences [7,9,15]. For example, in the monitoring of emergency events or of industrial sites, such as boilers, metallurgical furnaces, and automatic assembly lines, rapid data transmission enables the control center to adopt corresponding countermeasures to avoid disasters, while dragged data transmission will result in the control center not having adequate time to take measures to avoid disasters [10,31,52].

In summary, it is still a challenging issue in EHWSNs to design an optimal solar collector with minimal cost in order to optimize network performance. In the current study, the previous research mainly focused on the effective use of energy. However, there are few strategies that can optimize the network deployment cost at the same time, and further improve the network performance. Therefore, an energy conserving and transmission radius adaptive (ECTRA) scheme is proposed to make full use of energy, reduce delay, as well as network deployment costs for EHWSNs. The main contributions in this paper are listed as follows:

(1) An energy conserving approach is proposed to conserve energy and avoid outage for the nodes in hotspots, which are the bottleneck of the whole network. The novelty of this scheme is adaptively rotating the transmission radius. In this way, the nodes with the largest energy consumption in the network are rotated, balancing the energy consumption between the nodes and reducing the maximum energy consumption. Thus, the battery volume can be reduced, correspondingly, and the cost on hardware can be further reduced. In EHWSNs, in order to avoid outage for the nodes, energy harvesting hardware is designed according to the energy requirement of the node with maximum energy consumption when deploying the network. In general, nodes in hotspot areas consume the most energy, meaning that our battery volume should be designed to meet energy consumption of the nodes in hotspot areas. However, in fact, the energy consumption of most non-hotspot regional nodes is much smaller than the energy consumption of hotspot nodes, and the designed battery volume is much larger than the actual demand. Therefore, the above design results in substantial waste of hardware and energy and further increases the network cost. What is worse, the larger the difference in energy consumption between hotspot regional nodes and non-hotspot nodes, the more hardware and energy is wasted. Therefore, the most ideal solution is to make the energy consumption of the entire network uniform, so that the hardware and energy of each node can be fully utilized. Based on this, an energy conserving approach is proposed in this paper. The detailed idea is as follows: When the network sends data with a certain transmission radius, the energy consumption in the network is uneven. Thus, there is a region with largest energy consumption which determines the network lifetime. Considering the regions of maximum energy consumption are not coincident under different transmission radii, we propose to send data with different transmission radii, and then, the largest energy consumption after multiple radius transmitting data by turns is lower than the largest energy consumption using a single transmission radius. Thus, node requirements for energy harvesting hardware and network deployment costs can be reduced.
(2) The second innovation of the ECTRA scheme is that we only choose larger transmission radii than the previous radius to transmit data by turns, when the nodes can absorb enough energy from the surrounding environment. Then, the network performance can be further optimized in these two aspects: (a) Reduce network delay effectively. When the network uses a larger transmission radius, the hops in data transmitted to the sink needs are fewer, which can reduce delays effectively. (b) With larger radii, hotspot nodes near the sink area can conserve enough energy to transmit data when there is not sufficient energy. Thus, the battery volume or solar panel size can be reduced, and further, the cost can be reduced.
(3) After deep theoretical analyses, the results show the following advantages compared with the traditional method: (1) The ECTRA scheme can effectively reduce deployment costs. The analysis results show that the network performance is better than the previous strategy, even when the energy collection hardware cost is reduced by 29.58%; (2) The ECTRA scheme can effectively reduce network data transmission delay by 44–71% compared to the traditional method; (3) The ECTRA scheme shows a better balance in energy consumption, and the maximum energy consumption is reduced by 27.89%; (4) The energy utilization rate is effectively improved by 30.09–55.48%.

The rest of this paper is organized as follows: In Section 2, the related works are reviewed. The system model is described Section 3. In Section 4, a novel ECTRA scheme is presented. Performance analyses of ECTRA are provided in Section 5. We conclude in Section 6.

## 2. Related Works

This section is divided into three parts to discuss the three main works related to the research in this paper. The three works are listed respectively: (1) the related work on reducing and balancing energy consumption; (2) the related work on transmission radius, energy hole, and delay; (3) the related work on energy harvesting wireless sensor networks.

(1) The research on energy balance and energy hole-related issues [11,30,36]. In wireless sensor networks, since nodes are generally powered by a battery, the battery capacity of a node is generally limited, due to limitations in cost and size. Therefore, it is an important research content of designing to reduce energy consumption of nodes and extend the lifetime of network in wireless sensor networks (WSNs), and many strategies to reduce energy consumption are put forward. The research on reducing energy consumption can expand from each layer, such as, Medium Access Control (MAC) layer, network layer, application layer, and so on [2,7,8,9,10,11,12,13,37,38,39]. The operations of energy consumption in WSNs mainly have the following categories: sleep, sending data, receiving data, idle, computing, etc. Lots of research demonstrates that the main energy consumption in these operations comes from data communication, which includes sending and receiving data. In order to save energy, researchers have proposed duty cycle-based WSNs to reduce node energy consumption [7]. In this network, the time of the node is divided into fixed cycles, and in one cycle, the node is divided into two states: sleep and awake. In the state of sleep, the node turns off the wireless communication device to conserve energy [7]. However, this method increases the delay of event monitoring and data transmission. In general, the longer the time that a node is awake, the smaller the delay in event monitoring and data transmission in one cycle, and the larger the energy consumption. In short, the delay is inversely proportional to the length of awake time. Based on this, the research on how to minimize the time that nodes need to be awake and reduce delay, at the same time, is further expanded, and there are many strategies proposed. In the early studies, researchers used an optimized duty cycle, which reduced the time the node was awake, to save energy as much as possible when the application requirements were met [7]. In this way, the duty cycle in the whole network is the same, which contradicts the unbalanced requirement for the duty cycle, due to unbalanced data volume at different distances from the sink. Since the time that nodes need to wake is much more in the near-sink area, these nodes bear a large amount of data compared with the far-sink area nodes that bear a small amount of data. In other words, nodes in the far-sink area do not require such a big duty cycle, although it is an optimal duty cycle. To solve this problem, researchers put forward an adaptive duty cycle scheme to optimize network performance [7]. They propose to adaptively adjust the size of the duty cycle based on the amount of data the node is in charge of. When the amount of data undertaken by the node is large, a large duty cycle is adopted, and when the amount of data undertaken by the node is small, a smaller duty cycle is used, to save energy. However, this method seems reasonable in only some aspects. Considering that the network lifetime is determined by the node with the largest energy consumption in the network, this method cannot improve network lifetime, because it concentrates on reducing the energy consumption of nodes in the far-sink area, which is small for the small amount of data load. What is worse, decreasing the duty cycle of nodes in this area results in a worse delay in monitoring and data transmission. Chen et al., in ref. [53], noticed this problem and proposed a dynamic adaptive duty cycle scheme that is almost opposite to the above scheme. Near-sink area nodes with a large amount of data use the optimal duty cycle, and far-sink area nodes increase the time they are awake on this basis, because they have residual energy for a small amount of data load. Thus, it can speed up data transmission and reduce delay.

In summary, compared with the network that keeps the nodes awake, duty cycle-based WSNs can reduce energy consumption but this caused a longer delay at the same time. Thus, how to reduce the delay in duty cycle-based WSNs becomes an important issue. A duty cycle adjustment algorithm adaptively increasing the awake time to reduce the sleep delay effectively is proposed in ref. [53]. Sleep delay usually happens when sender nodes need to send data, and their next hop nodes are in sleep states. Obviously, synchronizing a sender with its next hop nodes is a feasible method to solve the above problem. When the sender needs to send data, its next hop nodes are in the awake state exactly, which can effectively reduce the sleep delay. Wu et al. [7] proposed a local coarse synchronization method based on the above method. Instead of requiring nodes in the entire network to synchronize, it allows nodes in the same area to be asynchronously awake and only requires each sender node to be basically synchronized with its next hop nodes. Furthermore, not requiring that each node is strictly synchronized and making only a part of the nodes maintain a loose synchronization is easy to implement. In this way, it is a perfect method to effectively reduce delay.

There are many schemes proposed to reduce energy consumption. Ref. [50] pointed out the complicated relationship between the energy consumption of transmitting unit bits by analyzing the relationship between the transmission power, the transmission success rate and the transmission distance of the node. In a wireless transmission environment, because the wireless signal is affected by all sorts of interference and signal attenuation, the receiver receives the data packet with a probability receiving relationship, instead of receiving successfully or not. Usually, when the distance between the sender and the receiver is close and the transmission power of sender is big, the receiver successfully receives the data with a high probability. Conversely, when the distance from the receiver exceeds a certain value, the acceptance rate of receiver drops sharply. The data transmission rate of the wireless link is defined as a probability less than 1. To ensure the accurate data transmission, certain strategies are taken to guarantee a good quality in wireless transmission with data loss, of which send-wait protocol is a simple and effective method [54]. There is a nonlinear relationship between the transmit power and the successful transmission rate of data, rather than a linear relationship. Therefore, finding a suitable transmission power that can minimize the energy required to successfully transmit unit-bit data is an important issue to study. Ref. [50] makes a further study and puts forward the algorithm for optimizing energy consumption across layers, which minimizes the energy required to successfully transmit unit-bit data and improve the energy utilization.

Further research expands upon the above study. An optimal transmission power minimizes the energy consumption per bit data. However, the transmit data node with the basic unit data packet includes an overhead, such as a data header, besides valid data. An effective way to reduce overhead, such as a data header, is to increase packet length, thus reducing the ratio between the header and the valid data. The problem is that a longer packet length may increase packet error rate, although it can reduce overheads like data header, because there is a certain loss and packet error rate in wireless transmission. Even worse, the increase of packet error rate will increase the numbers of packet retransmission, which will increase the overhead of the unit’s valid data instead. Therefore, Chen [55] et al. show the theoretical analysis results of the optimized packet size to minimize the energy consumption when the node transmits the valid data of the unit bits.

Reducing energy consumption is one of the most direct and effective methods to improve network lifetime, but is not an effective way. In the process of routing, some nodes chosen as relay nodes will die early for their higher energy consumption than other nodes. At this time, an important factor in improving energy efficiency is the balance in energy consumption of nodes. Thus, there are lots of energy consumption balanced routing algorithms proposed by researchers, as shown in refs. [27,28,30,31,39,41,52,56]. Summarizing some common features of the current energy consumption equilibrium routing strategies in ref. [56], we propose the design principle of this type of routing strategy. In fact, the key to the design principle of an energy consumption equilibrium routing strategy is to design what kind of cost function will choose the next hop node. When the energy consumption of a node is lower than the neighboring nodes of its period, if the characteristic of lower energy consumption can be reflected from the cost function, then the designed cost function can better guide the selection of the next hop, and thus, achieve a more balanced energy consumption.

(2) Related work on the transmission radius of the node, energy hole, and delay. The above research only concentrates on reducing energy consumption of the node. However, in fact, there is a particular phenomenon called energy hole [2,11,30,36], which is also seen in EHWSNs. Specifically, because the sink is the final node that other nodes transmit data to in the whole network, and the data of the node is generally routed to the sink through multi-hop relay in WSNs, this means that the node within one hop of the sink bears the data relay work of all other nodes in the network, and the energy consumption of nodes within one hop from the sink is much higher than that in other areas. Moreover, it is found that for normal WSN, the data amount of nodes within one hop from the sink bear more than 10 times the amount of data of nodes in the farthest area. Moreover, the energy consumption in this area is much higher than any other areas for its high data load, and thus, it is called a hotspot. When the energy in hotspots is exhausted, an area forms where all the nodes around the sink are dead, and this area is called an energy hole. The formation of the energy hole means the data of other nodes in the network cannot be forwarded to the sink, leading to early death of the network. However, at this time, there is lots of residual energy in the area whose distance from the sink is far (according to related research, up to 90% of energy is remaining in the network at this time). Obviously, this part of energy cannot be used effectively. An energy hole is formed due to the early death of nodes within one hop from the sink, so it has a close relationship with the transmission radius of the node. In general, when the transmission radius r is small, the energy consumption of the nodes in the near-sink area will be very high because they bear the data of the entire network, which causes early death of the network. Thus, increasing the transmission radius r can reduce the data load of nodes within one hop from the sink, and further decrease energy consumption in a certain area, which improves network lifetime. However, as the r increases, the energy consumption may not be reduced, although data load of the nodes decreases. This is because that the energy consumption of sending data from the node is proportional to the square or the fourth of the power of the transmission distance. When the transmission distance increases, the energy consumption of nodes will increase, although their data load is small [57,58]. Based on this, researchers expand the study on how to reduce maximum energy consumption in the network, and attenuate the impact of the energy hole. The general methods are as follows: (a) use an optimized transmission radius r to achieve an optimized lifetime; (b) send data of the nodes in a part of the non-hotspot area directly to the sink without routing through nodes near the sink to reduce high energy consumption in hotspot area, thus improving lifetime; (c) change the data collection mode. If each node sends data directly to the sink, the energy consumption of the far-sink area node is the highest, while that of the near-sink area node is the lowest. On the contrary, if multi-hop data routing to the sink method is taken, the energy consumption of the near-sink area node is the highest, while that of the far-sink area node is the lowest. Hence, researchers propose mixing these two data collection modes to balance energy consumption and further improve lifetime.

Transmission radius also has effects on the transmission delay of data. Since data packets are transmitted to the sink through multi-hop data transmission, the total delay of data transmission to the sink is the sum of each hop route delay, which indicates that increasing transmission radius can reduce delay. Of course, an optimal transmission radius should be gotten by considering delay, energy consumption, and lifetime together.

(3) Energy Harvesting Wireless Sensor Networks (EHWSNs). EHWSNs bring profound changes to WSNs whose energy is tense [39,40,41,42,43,44]. Since the sensor in EHWSNs increases hardware that can absorb energy, it can obtain energy from the surrounding environment. Theoretically speaking, sensor nodes can monitor the monitored objects for a long time without any guards. For example, solar-powered wireless sensor networks add a solar panel, rechargeable battery, and charge controller to normal sensor nodes. Among them, a solar panel is used to absorb solar energy and convert solar energy into electricity. A battery is used for storage and supply of power, and a charge controller is used to monitor the storage of the battery. When the battery power is below the threshold, the battery’s power supply appliance is disconnected to protect the battery from excessive power loss. The solar panel usually adopts photovoltaics (PVs), and its size determines how much energy is absorbed per unit time. In general, the larger the size of the solar panel, the more energy is absorbed per unit time, so the more electric power the nodes can use. However, the big size of solar panel needs a bigger construction cost. At the same time, the volume of sensor nodes is greater, which limits the deployment of the network and other aspects. Similarly with the solar panel, the bigger the battery is, the more electric power the nodes can conserve. In this way, when solar power is sufficient, the nodes can conserve enough energy to use when solar power is not sufficient. Of course, a big volume of battery requires a bigger construction cost. Therefore, reducing energy consumption is still a main goal in EHWSNs. Reducing the energy consumption of the node, effectively, can reduce the size of the solar panel and battery, thereby reducing deployment costs. Moreover, how to utilize absorbed energy is also a subject worth studying in EHWSNs. For example, in solar-powered WSNs, when the solar radiation is strong, the node can absorb a lot of energy through the solar panel, in addition to fully charging the battery’s electricity, and there is still surplus. In such cases, the key is to study how to make full use of energy that can be absorbed from the environment, rather than to reduce energy consumption. In summary, there are two essential points when studying EHWSNs: (a) minimize energy consumption; (b) utilize energy absorbed from the environment effectively.

The concept of reducing energy consumption of nodes is the same with normal WSNs, and what makes a difference in EHWSNs is the goal of reducing energy consumption. The main goal in WSNs is to increase lifetime as much as possible, while in EHWSNs it is to provide a benchmark for selecting the size of the solar panel and battery, in order to effectively reduce deployment costs. The design goal in EHWSNs is that the power supply system still has a high probability of providing energy normally in the harsh environment. For solar-powered wireless sensor networks, the power supply system should be designed to keep nodes working normally in the case of continuous low solar radiation intensity. If the energy consumption of nodes is low, then a smaller size of the solar panel and battery can be adopted, in order to reduce deployment cost.

When deploying the network, the suggestion of utilizing as much energy as possible is designed under the premise of being able to survive in a harsh environment. Therefore, most time in the network, the energy absorbed by nodes from the periodic environment is greater than the minimum energy required by the network. Thus, using this energy as much as possible to improve network lifetime is an important but challenging issue. The main difficulty is making full use of energy absorbed from environment when the energy is enough because the maximum energy that a node can utilize is designed according to the worst situation. Therefore, the main problems are summarized as follows: in solar-powered wireless sensor networks, there is enough solar energy to use at daytime, while no there is no solar energy to charge the electric power at night. Thus, the principle of energy utilization in such a system is storing as much energy as possible at daytime, in order to avoid the outage of nodes in the evening, or on continuously cloudy days when the solar energy is not sufficient. A universal law is that the solar energy gradually becomes stronger from morning to noon at 12:00, and then gradually weakens after 14:00. In order to make full use of energy, we can try to utilize as much energy as possible, even using the residual energy of battery when solar energy is going up. As time goes by in the morning, the solar energy will gradually increase, and it is enough sufficient to fully charge the battery. Therefore, if the energy is not used in advance at this time, when the battery is fully charged, the time for efficient use of energy will be very short. Another reason is that as time goes by in the afternoon, the solar energy is decreasing. Hence, at this time, we must reduce the previous higher energy consumption to maintain a high level of stored energy in the battery, so that it can be used in the evening or on continuously cloudy days. In short, it is suggested to use energy in advance when the radiation intensity of solar energy increases, and to conserve energy in advance to prepare for insufficient energy supplementation when the radiation intensity of solar energy decreases. What is worth noting is that the radiation intensity of solar energy is unpredictable in advance, because it is changes according to weather conditions and seasons. Here comes the conflict: if the energy is used in advance, the sudden weakening of solar energy due to a weather change may result in insufficient storage of the battery and possible outage. On the contrary, if a conservative “make ends meet” strategy is adopted, the energy may not be fully utilized. Thus, accurate prediction of solar energy has an important impact on improving energy efficiency in such EHWSNs. A considerable number of predictive models have been proposed by many researchers to model the energy harvesting process [41], such as the finite state Markov model and general stochastic model.

Although modeling the energy harvesting process can effectively predict the replenishment of solar energy, which helps to effectively design energy utilization strategies, in essence, the prediction of renewable energy for solar energy cannot be completely accurate. Also, when the prediction model is inaccurate, it may bring upon serious conditions of network outage. Therefore, the combination of solar energy supplemental prediction and adaptive energy utilization is an effective method. However, there are many situations involving network energy utilization, and efficient energy utilization is related to network performance, which makes the comprehensive optimization of EHWSNs very complicated. Obviously, there is still room for further research on efficient energy utilization in EHWSNs.

## 3. System Model

### 3.1. Network Model

In this paper, the network is deployed by 𝓍 sensor nodes in a circular area centered as a special node called sink with a radius R, according to ref [1,7,8,9,59]. Not considering data loss or error, all the generated data packets are transmitted directly or by relay nodes to the sink through multi-hop routing. The transmission radius of the node is r. The size of the data packet is set as f bytes, and the success rate per hop, which is called the reliability of the receiving node in this paper, is set as ζ.

### 3.2. Energy Harvesting Node Model

**Figure 1:**
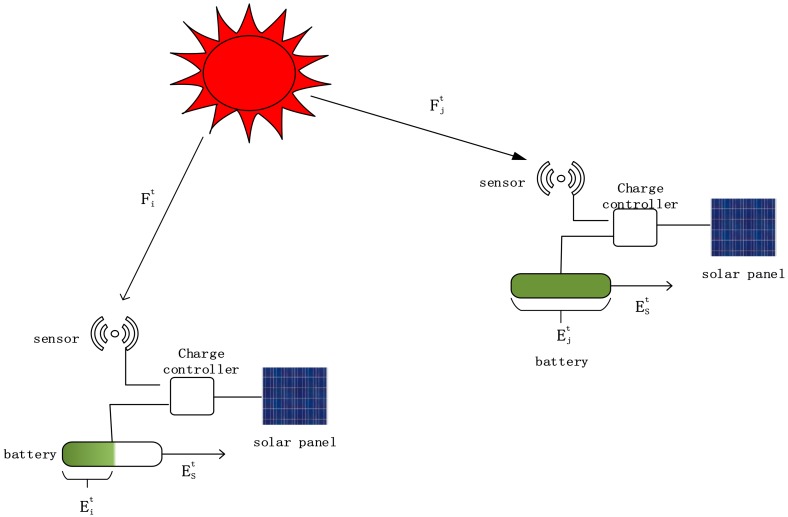
Solar sensor node structure.

In EHWANs, the sensor node mainly consists of five parts: a processor and sensor module, a wireless communication module, a solar collector, a battery, and the power controller. Combined with the energy harvesting model in ref [39,40,43,44,60], the structure in this paper is shown in Figure 1.

We define $E_{i}^{t}$ as the energy level of the node i at time t, ${ℱ}_{i}^{t}$ as the solar radiation on the node i at time t, and as the energy consumption of the node i. ℬ represents the battery volume of the node [39]. We assume that the solar energy received by each node at the same time is the same, in other words, the influence of positional factors on the absorption of solar energy is ignored [39]. Then, the equation of calculating the residual energy $E_{ri}$ of the node i is given as follows:

(1) $$\;E_{ri\;}^{t}=\min\left\{\max\{0,E_{i}^{t-1}-E_{s}^{t}+{ℱ}_{i}^{t-1}\right\},ℬ\}$$

### 3.3. Energy Consumption Model

Like the general energy consumption models for WSNs [50,61], the energy consumption is mainly determined by the transmission power and data packets that nodes need to send and receive. Moreover, it is also affected by the time duration of transmission [31]. According to this, we give the calculation of energy consumption next.

Here, 𝓋 is the data rate (in bits/s) and calculated with the value of 2400 bits/s. $D^{S}$ is the sending data volume of node and $D^{R}$ is the receiving data volume of node. f is the size of a data packet (in byte). $P_{T}\;$ and $P_{R}$ are the transmission power and receiving power of the node, respectively [50,61].

The energy consumption of sending data packets is calculated by the following equation:

(2) $$\;E^{s}=P_{T}\cdot f\cdot D^{S}/𝓋\;$$

The energy consumption of receiving data packets is calculated by the following equation:

(3) $$\;E^{R}=P_{R}\cdot f\cdot D^{R}/𝓋\;$$

The total energy consumption of the current node is calculated by the following equation:

(4) $$\;E^{total\;}=E^{s}+E^{R}=f\cdot (P_{T}\cdot D^{S}+P_{R}\cdot D^{R})/𝓋$$

In the network with a given reliability valuing ζ, the transmission power of the node is determined by the transmission radius and data packet, which can be calculated by Equation (23). In our subsequent research process, in order to facilitate the subsequent calculations and unit unification, here, we convert the power unit from dBm to mw.

(5) $$\;P={10\;}^{\frac{P\left(in\;dBm\right)}{10}}$$

According to the path loss model [50,61], we define d as the distance from the sending node to the receiving node, which is called the transmitting distance, and 𝓈 as the path loss coefficient. $L_{d_{0}}$ indicates the path loss when the distance between the sending node and the receiving node is a reference distance $d_{0}$, here $d_{0}=1\;m$ [50,61]. $X_{\sigma }\sim N\left(0,\sigma \right)$ is a Gaussian random variable with zero mean and standard deviation σ in dB. Thus, we can calculate the receiving power by the following equation:

(6) $$\;P_{R}(d)\;=P_{T}-10𝓈log_{10}\left(\frac{d}{d_{0}}\right)-L_{d_{0}}+X_{\sigma }$$

### 3.4. Problem Statement

The optimization of an EHWSN can be characterized by several performance indicators as explained below.

(1) Minimize deployment costs

In EHWSNs, the electricity of the battery reflects the maximum chargeable capacity of the node. In general, the larger the battery capacity is, the more favorable the node is, because the larger battery capacity can absorb and conserve more energy than a small battery when the surrounding environment is rechargeable. But the problem is often not so simple. Based on the consideration of construction cost, the large battery capacity will require higher construction costs.

Generally speaking, when the battery capacity is large, the solar collector’s solar panel area is also large, and the power controller function also needs to be more powerful, and vice versa. Therefore, we can use one of these three components to reflect how much the construction cost of the node will be. In this paper, battery power is selected to reflect the node construction cost. Consuming the battery power of node i is ${𝓋}_{i}$, and the mapping function of the cost of energy harvesting hardware is ℱ, then the cost of energy harvesting hardware of node i is $ℱ\left({𝓋}_{i}\right)$. Thus, the hardware cost of energy collection in the entire network is $\overset{n}{\underset{i=1}{\sum }}ℱ\left({𝓋}_{i}\right)$. Above all, our goal of minimizing network deployment costs can be expressed as follows:

(7) $$min\left(C_{1}\;\right)=min(\sum_{i=1}^{n}ℱ\left({𝓋}_{i}\right)).$$

(2) End-to-sink delay ($T_{sink}^{d}$)

End-to-sink delay represents the time that a node with a distance d m from the sink needed when sending data to the sink, and data is received successfully by the sink, and it can be calculated by the equation $T_{sink}^{d}=\overset{h}{\underset{i=0}{\sum }}T_{i}^{d}$, where h is defined as the hops that data need to route though until data is sent to the sink, and $T_{i}^{d}$ indicates the time of the $i^{th}$ hop. In general, reducing the end-to-sink delay is better for network performance. We can conclude this relation by the following equation as our study goal:

(8) $$\;min\left(T_{sink\;}^{d}\right)=min\left(\overset{h}{\underset{i=0}{\sum }}T_{i}^{d}\right)$$

We assume that the time interval of each node in the network transmission is the same, then the number of hops can be used to represent the end-to-end delay to simplify our calculation more directly, which can be expressed as follows:

(9) $$\;min\left(T_{sink\;}^{d}\right)=min\left(h\right)$$

(3) Maximizing energy utilization

Energy utilization is the percentage of energy efficient use of the total energy, which is a comprehensive indicator for measuring the level of energy utilization by technology and economic performance. In this paper, energy utilization is the ratio of the energy consumed by the network to the available energy of the network within an hour, as shown in Equation (10):

(10) $$\;U=\frac{E_{j}\;}{A_{j}}$$

where j is the j-th node in the network, $E_{j}$ indicates the energy consumption of the node j within an hour, and $A_{j}$ is set as the available energy within an hour. If 𝕟 is the total number of nodes in the network, then the energy utilization in the entire network is

(11) $$U=\sum_{j=1}^{𝕟}E_{j}/\sum_{j=1}^{𝕟}A_{j}.$$

The maximization of network energy utilization will improve the effective use of network energy, so our goal is focused on making the ratio of energy consumed to the available energy in the network largest as far as possible, which is shown in the following:

(12) $$\max{(}_{U})=\max(\sum_{j=1}^{𝕟}E_{j}/\sum_{j=1}^{𝕟}A_{j}).$$

(4) Network lifetime

In EHWANs, as long as the remaining battery power of the sensor node is higher than the minimum $v_{\nabla }$ at any time, the node will not die, that is expressed in the following formula.

(13) $$v_{i}\geq v_{\nabla }\;|\forall t,i\;$$

In summary, the goal of this paper can be stated as following Equation (14):

(14) $$\;\{\begin{array}{c} min\left(T_{sink\;}^{d}\right)=min\left(\overset{h}{\underset{i=0}{\sum }}T_{i}^{d}\right)\\ \max{(}_{U})=\max(\sum_{j=1}^{𝕟}E_{j}/\sum_{j=1}^{𝕟}A_{j})\\ min\left(C_{1}\right)=min\left(\overset{n}{\underset{i=1}{\sum }}ℱ\left({𝓿}_{i}\right)\right)\\ s.t.\;\;v_{i}\geq v_{\nabla }\;,\;\forall t,i \end{array}$$

## 4. The Design of the ECTRA Scheme

### 4.1. Research Motivation

The research motivation is firstly given in this section. There are two innovations of the ECTRA scheme: (1) Reduce the maximum energy consumption by adjusting the transmission radius and using them by turns, in order to decrease battery volume and the size of the solar panel when designing the network, thus reducing deployment cost. (2) Make full use of energy absorbed in the network by sending data with a bigger transmission radius when the solar radiation is strong enough. On the one hand, it improves energy efficient utilization for making full use of the energy that nodes absorb. On the other hand, it reduces delay because increasing transmission radius decreases the incidence of hops in data transmission.

**Figure 2:**
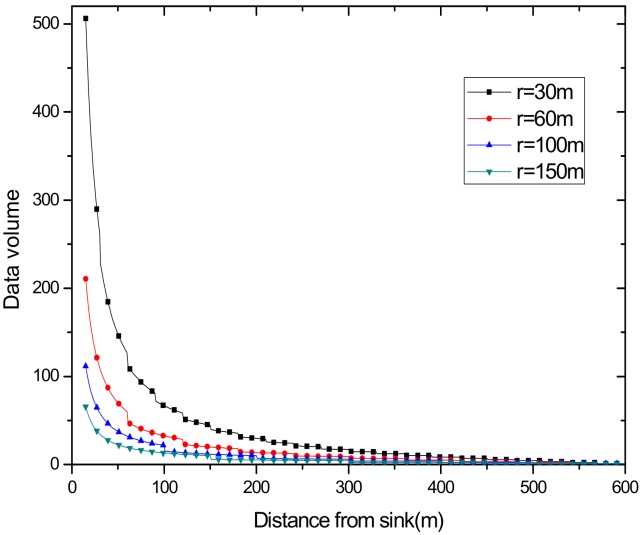
Data volume of nodes.

**Figure 3:**
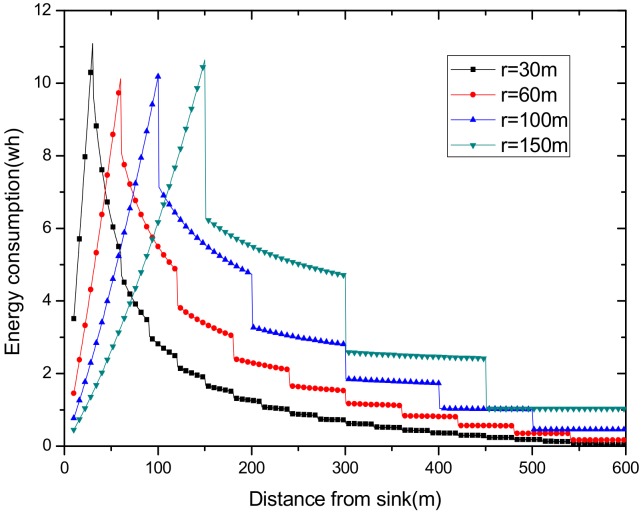
Energy consumption using fixed transmission radius.

(1) Reduce the maximum energy consumption in the network. Figure 2 shows the data amount the nodes bear in the network. A universal law can be seen from the figure that the amount of data undertaken by the near-sink area node is much larger than that of the far-sink area node. As the transmission radius becomes bigger, the data amount of the node at the same distance from the sink becomes less and less. The most extreme case is that when the transmission radius is equal to the network radius, each node sends data to the sink directly without any relay, which means the amount of data each node bears is 1. Figure 3 shows energy consumption of nodes at different distances from the sink in the network. Obviously, the law of energy consumption of nodes is different from the law of the amount of data undertaken. The energy consumption of a node is proportional to the transmission power required by the node, and the amount of data it undertakes. Nodes near the sink require a small transmission power while nodes far from the sink require a big transmission power. Analyzing Equation (5), due to the attenuation of the wireless channel, as the transmission distance increases, the transmission power needs to be sharply increased to ensure a certain reception rate of the receiver. Therefore, energy consumption of the node whose distance from the sink is within the transmission radius r is low for its small transmission power, although it bears a large amount of data. However, as the distance increases, the transmission power growth rate is much faster than the data volume reduction rate, causing energy consumption to increase until the distance reaches r. As for nodes whose distance from the sink is greater than r, their transmission power is the same for the same transmission distance. Since their data load decreases, energy consumption gradually decreases.

**Figure 4:**
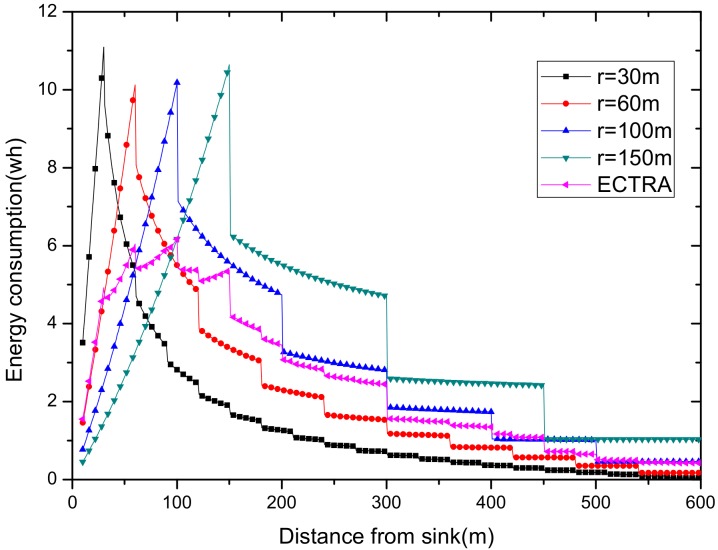
Energy consumption rotated by several transmission radius.

**Figure 5:**
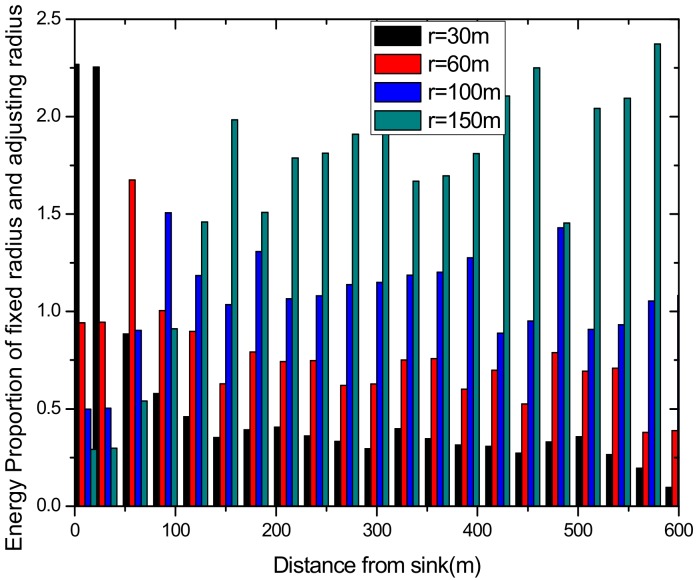
Energy proportion of fixed radius and adjusting radius.

As can be seen from Figure 3, for a network with a transmission radius r, the nodes of maximum energy consumption locate at the distance r from the sink. As common sense, network lifetime depends on the maximum energy consumption in the network. In this way, when the node’s transmission radius r is determined, the network lifetime is also determined. In further research, we find that the location of the node with maximum energy consumption is different for the different transmission radii. An inspiration came to mind: if the network is rotated with different sending radii at different times, then the highest energy consumption is taken by different nodes in turn. After rotations with several transmission radii, the maximum energy consumption in the network can be reduced sharply, compared with the fixed transmission radius (as shown in Figure 4). Figure 5 shows this relationship more clearly by calculating the ratio of energy consumption with the fixed radius to energy consumption of the radius after adjusting. Our data for solar radiation are selected from the data of the Solar Radiation Laboratory of Texas, TX, USA [62]. Moreover, energy consumption corresponds to the size of the battery storage capacity, as well as the size of the solar panel. Large energy consumption requires a large battery capacity and solar panel area, while small energy consumption can reduce battery capacity and the solar panel area, which can reduce costs. It can be considered that the proportion of node consumption is positively related to the ratio of cost savings, so it can be approximated that costs can be saved in a corresponding ratio for the battery to that of the reduction in energy consumption.

**Figure 6:**
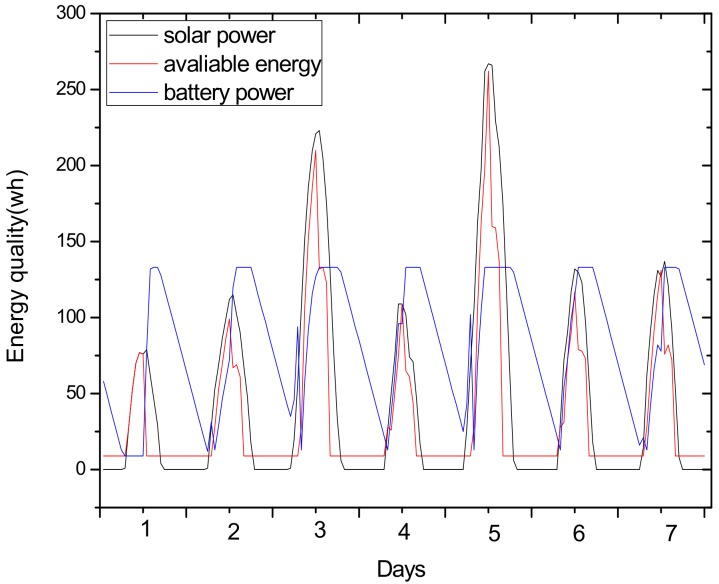
Solar radiation, available energy and battery power in seven days.

**Figure 7:**
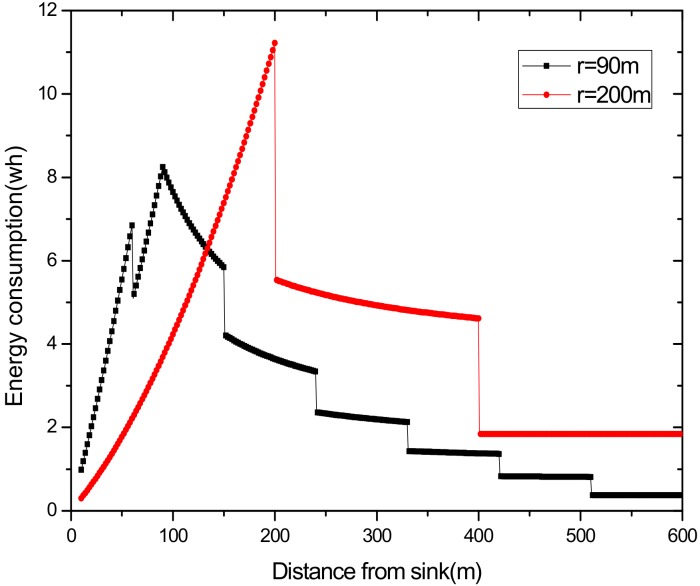
Comparison of energy consumption with a small and big transmission radius.

**Figure 8:**
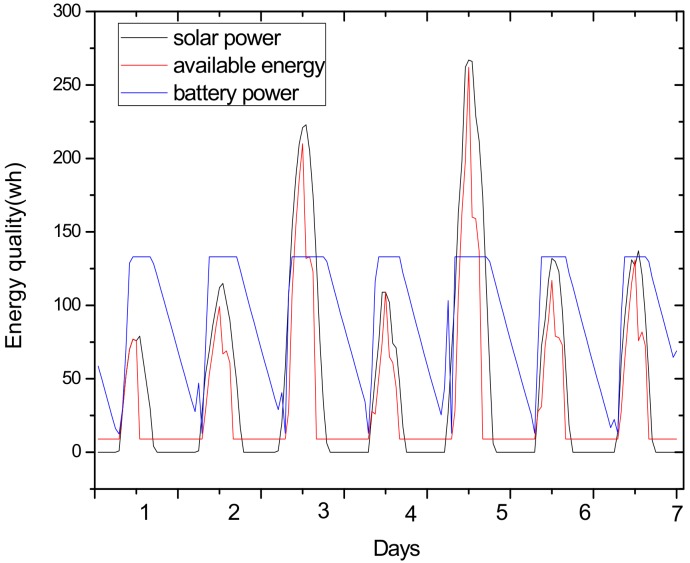
Battery power using bigger transmission radius.

**Figure 9:**
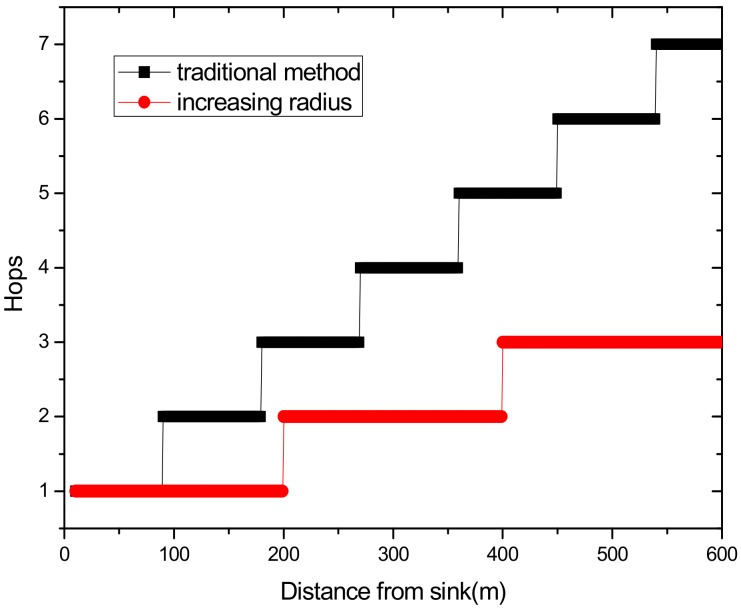
Comparison of delay.

(2) Make full use of energy absorbed in the network by sending data with a bigger transmission radius to reduce delays and improve network performance. How to reduce energy consumption is discussed above; next, we will discuss the second innovation in this paper. Besides energy consumption, another major feature is absorbing solar energy in a solar-powered wireless sensor network. How to use absorbed solar energy effectively in order to improve network performance is a hot topic. Figure 5 gives a typical example of the relationship between storing energy in a battery, the available energy of nodes, and solar radiation intensity, within 7 days. Figure 6 shows the relationship between the maximum available energy nodes can use, solar energy, and battery power, in 7 consecutive days, where nodes consume energy at a value of the available energy at night (the battery volume is 133 wh and its initial energy is 67 wh). Since outage of the nodes in harsh surroundings is taken into account by the power supply system, when the solar energy is sufficient enough, and especially, the battery’s electrical energy is fully conserved, there is still a portion of the available energy that is not being fully utilized. Here, sending data with a bigger transmission radius is an effective solution. As shown in Figure 7, in a network with radius R=600 m, energy consumption with transmission radius r=200 m is larger than that with r=90 m in most areas. We adopt a bigger transmission radius in the network and calculate the battery power. As can be seen from Figure 8, when the energy is sufficient, the larger transmission radius makes full use of the energy, which increases the energy that can be utilized without affecting other network performance. Furthermore, increasing transmission radius reduces network delay, which is clearly indicated in Figure 9.

### 4.2. The Design of ECTRA Scheme

#### 4.2.1. The Calculation of Data Volume and Transmission Power in the Network

In this section, the formulas of data load and energy consumption of nodes in the network are given at first. The transmission radius of nodes is noted as *r*. If the distance from the node to the sink point is less than *r*, the node transmission distance is its distance from the sink, otherwise, its transmission distance is *r*. To ensure reliable transmission, in the network with reliability ζ, the number of retransmissions is 1/ζ [60].

**Theorem** **1.** *In EHWSNs, the network radius is set as* R *, the node transmission radius is* r *, and ensure data transmission reliability is* ζ *. Considering a node whose distance from the sink is* d *, the data volume it needs to transmit is calculated by the following equation:*

(15) $$\;D_{d}=\left(1+z+\frac{r}{d}\overset{z}{\underset{i=1\;}{\sum }}\frac{i}{\zeta^{i}}\right)\beta \;,\;z=\lfloor \frac{R-d}{r}\rfloor$$

**Figure 10:**
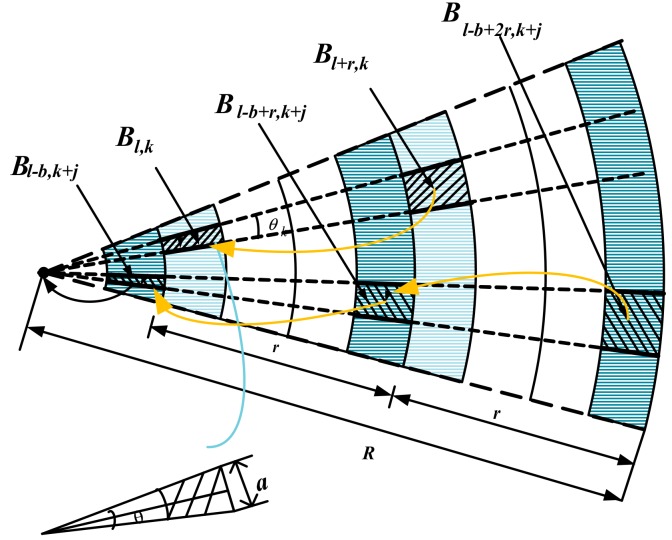
Routing process for data aggregation.

**Proof.**  We divide the arc area into many small arcs, and in this way, we can regard every shaded area as the rectangle. Figure 10 shows the routing process for data aggregation. ☐

Considering the amount of data that the sector ring area of width a assumes, where the distance of the node n from sink is l. Then, the sector ring must bear the forwarding of data from the sector ring with a width of a at l+r, l+2r…l+zr. If a is very small, it can be considered that the amount of data borne by each node in this area is the same.

As can be seen from the Figure 10, if the width of the rectangle is fixed, then its length will be a little larger as the distance increases from l to l+r, l+2r…l+nr. To calculate this changing length, we consider using the length of the first shaded area whose distance form sink is d to define the length in the rear. The details are as follows:

Defining the length of the first shaded area as b, then, in a triangle with an angle of $\frac{\theta }{2}$:

(16) tanθ2=b2l=b2l.

We can calculate the θ is

(17) $$\theta =2arctan\frac{b}{2l}.$$

As the node whose distance from sink is l+ir, define the length of its area as $b_{i}$, then area of the region can be calculated by

(18) $$S_{i}=a\times b_{i}=a\times 2\times \left(l+ir\;\right)\times tan\left(2arctan\frac{b}{2d}\right).$$

The number of nodes in the area where the node is located is

(19) $$N_{i}=\rho S_{i}=\rho ab_{i}=\rho \times a\times 2\times \left(l+ir\right)\times tan\left(2arctan\frac{b}{2l}\right),\;i\leq \lfloor \frac{R-l}{r}\rfloor .$$

The probability of each node generating data is β, the sector ring where the node is located must bear the forwarding of the node data with the sector ring width a at $l+r,l+2r,\ldots l+zr,z=\lfloor \frac{R-l}{r}\rfloor .$ Then, the amount of data that needs to be forwarded in this area is

(20) $$D_{i}=\rho S_{i}\beta ,\;i\leq \lfloor \frac{R-l}{r}\rfloor .\;$$

Consider that the number of retransmissions is 1/ζ in a network with reliability ζ. The node whose distance from sink is l+ir, and we can calculate that its actual data volume is

(21) $$D_{i}=\rho S_{i}\beta {\left(\frac{1}{\zeta }\right)}^{i+1},\;i\leq \lfloor \frac{R-l}{r}\rfloor .$$

Therefore, the data volume borne by the area where node n is located is

(22) $$D_{n}=\sum_{i=0}^{z}\rho S_{i}\beta {\left(\frac{1}{\zeta }\right)}^{i+1},\;z=\lfloor \frac{R-l}{r}\rfloor .$$

If a is very small, it can be considered that the amount of data borne by each node in this area is the same. Then, the borne data volume of this node, whose distance from the sink is l, is calculated as follows:

$$\begin{array}{l} \;D_{l}=\frac{d_{n}}{d_{0}}=\frac{\sum_{i=0}^{z}\rho S_{i}\beta {\left(\frac{1}{\zeta }\right)}^{i+1}}{\rho S_{0}{\left(\frac{1}{\zeta }\right)}^{1}}=\frac{\sum_{i=0}^{z}S_{i}\beta {\left(\frac{1}{\zeta }\right)}^{i+1}}{S_{0}{\left(\frac{1}{\zeta }\right)}^{1}}\\ \;=\frac{2\tan\left(2arctan\frac{b}{2l}\right)al\beta {\left(\frac{1}{\zeta }\right)}^{1}+2\tan\left(2arctan\frac{b}{2l}\right)a\left(l+r\right)\beta {\left(\frac{1}{\zeta }\right)}^{1+1}+\cdots +2\tan\left(2arctan\frac{b}{2l}\right)a\left(l+zr\right)\beta {\left(\frac{1}{\zeta }\right)}^{z+1}}{2\tan\left(2arctan\frac{b}{2l}\right)al{\left(\frac{1}{\zeta }\right)}^{1}}\\ \;=\left(1+\frac{l+r}{l}\left(\frac{1}{\zeta }\right)+\cdots +\frac{l+zr}{l}{\left(\frac{1}{\zeta }\right)}^{z}\right)\beta =\left(1+z+\frac{r}{l}\left(\frac{1}{\zeta }\right)+\frac{2r}{l}{\left(\frac{1}{\zeta }\right)}^{2}+\cdots +\frac{zr}{l}{\left(\frac{1}{\zeta }\right)}^{z}\right)\beta \\ \;=\left(1+z+\frac{r}{l}\overset{z}{\underset{i=1\;}{\sum }}(i{\left(\frac{1}{\zeta }\right)}^{i})\right)\beta =\left(1+z+\frac{r}{l}\overset{z}{\underset{i=1}{\sum }}\frac{i}{\zeta^{i}}\right)\beta ,\;where\;z=\lfloor \frac{R-l}{r}\rfloor \end{array}$$

**Figure 11:**
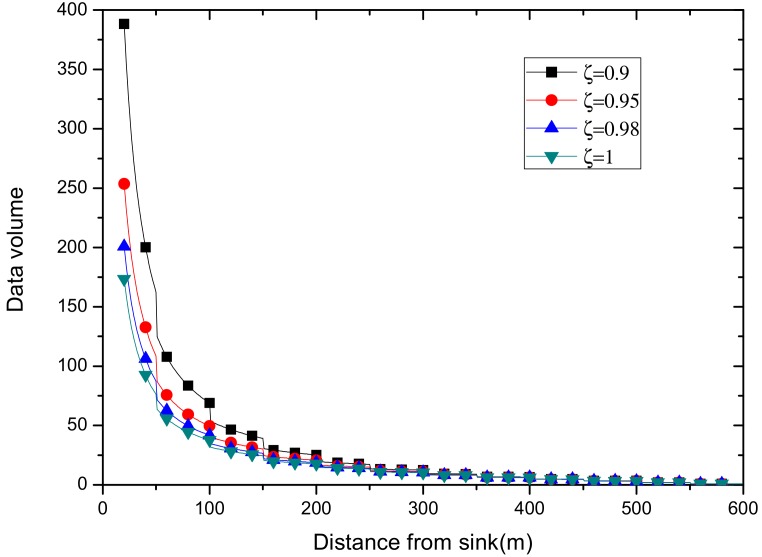
Data volume at different reliabilities.

According to above formula, the data volume of the nodes from sink at different distances is calculated with the transmission radius r=50 m under a reliability which is 0.9, 0.95, 0.98, 1, respectively. From Figure 11, we can find that the overall curve obeys the trend of decreasing with increasing distance from the sink. Obviously, reliability influences the data volume, and the bigger the reliability is, the smaller the data volume is.

This also demonstrates that no matter how great is the reliability the data is transmitted with, the overall trend of the amount of data changing with distance cannot be changed, that is, the amount of data transmitted by the node near the sink point is large, but small by the node, in far-sink areas.

**Theorem** **2.** *In order to ensure the reliability of transmission between nodes, in a network with reliability* ζ *, the transmission power of the node with transmission radius* r *is calculated as follows:*

(23) $$\;P_{T}=10𝓈log_{10\;}\left(\frac{d}{d_{0}}\right)+L_{d_{0}}+X_{\sigma }+𝓅-2\ln\left[2\left(1-\zeta^{\frac{1}{8f}}\right)\right]\times \frac{𝓋}{𝒷}$$

**Proof.**  We know that the packet acceptance rate (ζ) is used to measure the quality of the communication link, and it is influenced by SNR (signal-to-noise ratio), which is a main technical indicator for measuring the reliability of communication system quality. SNR (signal-to-noise ratio) is a parameter that describes the proportional relationship between the active component and the noise component in the signal, which has different specific definitions in different application areas. A common calculation is the ratio of the power of the active component in the signal to the power of the noise component. The following equation is given to express their relation:

(24) $$\;\zeta_{d}={\left(1-\frac{1}{2}exp^{-\frac{SNR_{d}\;}{2}\times \frac{𝒷}{𝓋}}\right)}^{8f}$$

Here, *d* is the distance between sending node and receiving node, 𝓋 is the data rate, f is the size of a data packet (in byte), and 𝒷 is noise bandwidth, which is valued as 30 kHz. ☐

(25) $$SNR=10log_{10\;}\left(\frac{P_{R}}{𝓅}\right)$$

𝓅 depends on the wireless signal and environment. According to the literature, the background noise value of the general network environment is −115 dBm.

The path loss is modeled as a log-distance path model, which follows a normal distribution in dB as described in ref [50,61,63]. Defining the path loss with a distance between two nodes valuing $d_{0}\left(m\right)$ as the reference distance, then, the path loss with a distance of d(m) can be calculated by the following equation:

(26) $$\;L_{d}=10𝓈log_{10\;}\left(\frac{d}{d_{0}}\right)+L_{d_{0}}+X_{\sigma }$$

Here, $X_{\sigma }$ is a random process, which means a function of time. If not assuming a dynamic environment, we use it as a constant random variable to model a specific link with time.

According to the path loss model, if we define $P_{R}$ as the transmission power in dBm, the receiving power of the node is

(27) $$P_{R}\left(d\right)=P_{T}-10𝓈log_{10}\left(\frac{d}{d_{0}}\right)-L_{r_{0}}+X_{\sigma }.$$

Considering that the unit of 𝓅 is dBm, we can get the formula

(28) $$SNR_{d}=P_{RdB}\left(d\right)-𝓅=P_{T}-10𝓈log_{10}\left(\frac{d}{d_{0}}\right)-L_{d_{0}}+X_{\sigma }-𝓅.$$

Hence, combined with equation, we get the relationship with reliability and transmission radius, as follows:

(29) $$\;P_{T}=10𝓈log_{10\;}\left(\frac{d}{d_{0}}\right)+L_{d_{0}}+X_{\sigma }+𝓅-2ln\left[2\left(1-\zeta^{\frac{1}{8f}}\right)\right]\;\times \frac{𝓋}{𝒷}$$

**Figure 12:**
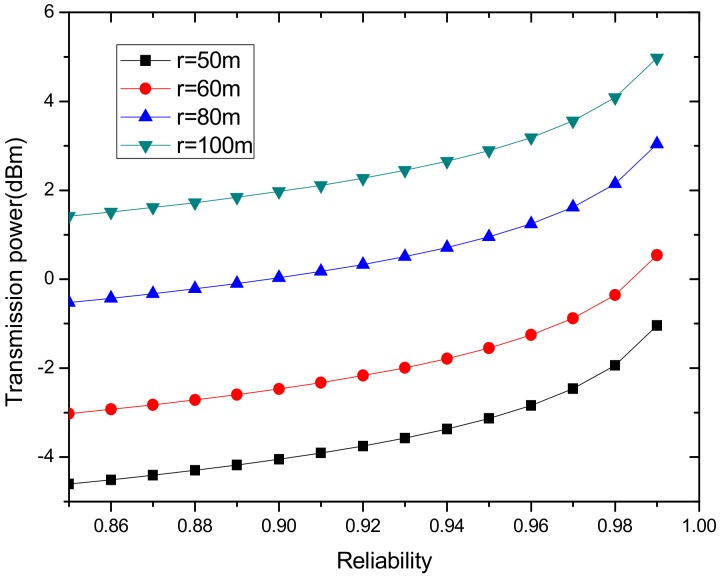
The transmission power under different reliabilities.

**Figure 13:**
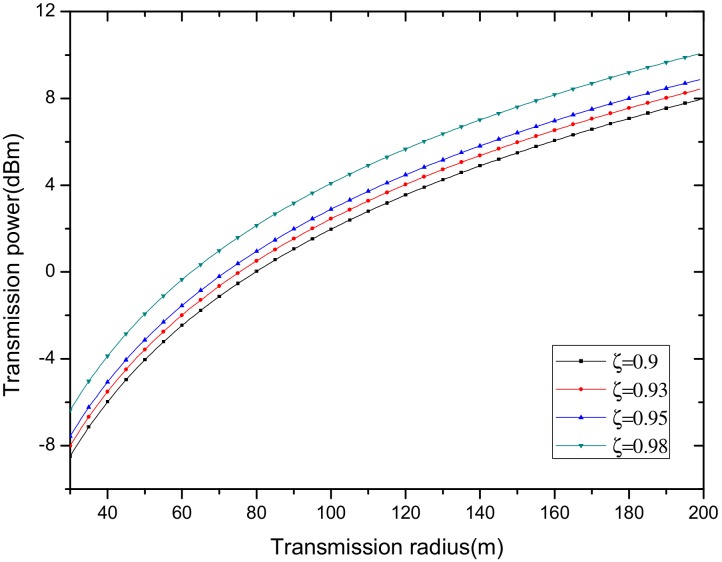
The transmission power under different transmission radii.

In order to further explore the relationship between transmission radius and power and reliability according to the above formula, we draw Figure 12 and Figure 13.

As can be seen from the above Figure 12 and Figure 13, the transmission power is closely related to the acceptance rate of the receiving node and transmission radius. For the same reliability, a larger transmission radius requires a larger transmission power. When the transmission distance is the same, the greater the acceptance rate of the receiving node is, the higher the transmission power that is required. Therefore, in order to ensure high reliability, the transmission power is required to be large, and for the same reliability, the transmission power is required to be higher when the transmission radius is larger.

#### 4.2.2. The Design for Adaptive Adjustment Transmission Radius Algorithm

Having disserted the core idea of ECTRA scheme, in this section, we will give the dynamic adaptive transmission radius adjustment algorithm. From the above theoretical analysis, when the transmission radius is r, the node with the largest energy consumption appears at the distance r from the sink. A main idea of the ECTRA scheme is selecting a set of optimized r sets, such as $\Upsilon =\left\{r_{0},r_{1},r_{2}\ldots r_{n}\right\}$, in order to reduce the maximum energy consumption as much as possible, and further reduce the cost of energy absorbing hardware (such as solar panels and battery capacity). Specially, when adopting different transmission radii $r_{i}$ in the network, the node with the largest energy consumption appears in different positions. Therefore, after multiple r rotations, the maximum energy consumption of the network is lower than the maximum energy consumption of any single r.

Therefore, the key of the algorithm in this section is how to find a set of optimized Υ sets to minimize the largest energy consumption. Here, a full search algorithm is adopted in this paper. For any given network, assume the original transmission radius as r. The algorithm in this paper searches for a set of transmission radii near r with a variable quantity ${𝓍}_{\nabla }^{i}$ (${𝓍}_{\nabla }^{i}$ that indicates the variable quantity used in the $i^{th}$ search for optimal r set). The formed r set searched in a variable quantity ${𝓍}_{\nabla }^{i}$ from the initial transmission radius r is recorded as $\Upsilon =\left\{r+{𝓍}_{\nabla }^{i},r-{𝓍}_{\nabla }^{i},\;r+2{𝓍}_{\nabla }^{i},r-2{𝓍}_{\nabla }^{i},\;r+3{𝓍}_{\nabla }^{i},r-3{𝓍}_{\nabla }^{i},\;\ldots ,r+\kappa {𝓍}_{\nabla }^{i},r-\kappa {𝓍}_{\nabla }^{i}\;\right\}$. During each search with variable quantity ${𝓍}_{\nabla }^{i}$, calculate the maximum energy consumption after different transmission radius rotations are changed with ${𝓍}_{\nabla }^{i}$. If the maximum energy increases after adding a new transmission radius $r\mp j{𝓍}_{\nabla }^{i}$, then stop the search for the current variable quantity ${𝓍}_{\nabla }^{i}$, and record the maximum energy consumption $E_{max}^{i}$ after rotating with the current transmission radius set adjusted by the $i^{th}$ variable quantity ${𝓍}_{\nabla }^{i}$ and the number of rotations $S_{i}$. Then, continue the above process by changing a new variable quantity ${𝓍}_{\nabla }^{i}$ until ${𝓍}_{\nabla }^{i}$=$\frac{r-\varrho }{2}$, in which ϱ is the distance of the first node from the sink. The last variable quantity is ${𝓍}_{\nabla }^{m}$. In this way, a group of maximum energy consumption $E_{max}=\left\{E_{max}^{1},E_{max}^{2},E_{max}^{3},\ldots E_{max}^{j},\;\ldots E_{max}^{m}\right\}$ and numbers of rotation $S=\left\{S_{1},\;S_{2},\;\ldots .,\;S_{m}\right\}$ are formed after m searches. Assuming the minimum value in $E_{max}$ as $E_{max}^{𝓀}$, then we can get a group of optimal transmission radii:$\;\Upsilon =\left\{r+{𝓍}_{\nabla }^{m},r-{𝓍}_{\nabla }^{m},\;r+2{𝓍}_{\nabla }^{m},r-2{𝓍}_{\nabla }^{m},\;r+3{𝓍}_{\nabla }^{m},r-3{𝓍}_{\nabla }^{m},\;\ldots ,r+S_{𝓀}{𝓍}_{\nabla }^{m},r-S_{𝓀}{𝓍}_{\nabla }^{m}\;\right\}$.

The specific Algorithm 1 is given below. The meaning of signals used in Algorithm 1 are

${𝓍}_{\nabla }^{i}$: the variable quantity of the transmission radius that can increase or decrease in the $i^{th}$ search
${𝓍}_{\nabla }^{ini}$ is an initial variable quantity of transmission radius
$\Delta_{x}\;$is the increasing value of ${𝓍}_{\nabla }^{ini}$
$E_{d}^{r}$ is the energy consumption at the distance d with the transmission radius r
$\Upsilon_{o}=\left\{r+{𝓍}_{\nabla }^{o},r-{𝓍}_{\nabla }^{o},\;r+2{𝓍}_{\nabla }^{o},r-2{𝓍}_{\nabla }^{o},\;r+3{𝓍}_{\nabla }^{o},r-3{𝓍}_{\nabla }^{o},\;\ldots ,r+S_{𝓀}{𝓍}_{\nabla }^{o},r-S_{𝓀}{𝓍}_{\nabla }^{o}\;\right\}$ is a set of optimal transmission radii
$E_{max}=\left\{E_{max}^{1},E_{max}^{2},E_{max}^{3},\ldots E_{max}^{j},\;\ldots E_{max}^{m}\right\}$, in which $E_{max}^{j}$ indicates the maximum average energy consumption after rotating with transmission radius set$\;\Upsilon =\left\{r+{𝓍}_{\nabla }^{j},r-{𝓍}_{\nabla }^{j},\;r+2{𝓍}_{\nabla }^{j},r-2{𝓍}_{\nabla }^{j},\;r+3{𝓍}_{\nabla }^{j},r-3{𝓍}_{\nabla }^{j},\;\ldots ,r+\kappa {𝓍}_{\nabla }^{j},r-\kappa {𝓍}_{\nabla }^{j}\;\right\}$ at the $j^{th}$ variable quantity ${𝓍}_{\nabla }^{j}$, and $E_{max}$ indicates the set of maximum energy consumption after rotating with variable quantity$\;\left\{{𝓍}_{\nabla }^{1},\;{𝓍}_{\nabla }^{2},\;{𝓍}_{\nabla }^{3},\ldots .{𝓍}_{\nabla }^{m}\right\}$
$E_{max}^{o}=min\left\{E_{max}^{1},E_{max}^{2},E_{max}^{3},\ldots E_{max}^{j},\;\ldots E_{max}^{m}\right\}$ is the maximum energy consumption of an optimal transmission radius set
r is a given transmission radius, *R* is network radius, and ϱ is the distance of the first node from sink. ζ is the reliability in the transmission.

**Algorithm 1.** Search the optimal transmission radius set algorithm.INPUT: r, *R*, ϱ, ζ,$\;{𝓍}_{\nabla }^{ini}$, $\Delta_{x}$.OUTPUT: $\Upsilon_{o}=\left\{r+{𝓍}_{\nabla }^{o},r-{𝓍}_{\nabla }^{o},\;\;r+2{𝓍}_{\nabla }^{o},r-2{𝓍}_{\nabla }^{o},\;\ldots ,r+S_{𝓀}{𝓍}_{\nabla }^{o},r-S_{𝓀}{𝓍}_{\nabla }^{o}\;\right\}$, $E_{max}^{o}$ 1: set $i=0,\;j=1,\;\Upsilon_{0}=\left\{r\right\},$
ℵ=1, μ=1 2: ${𝓍}_{\nabla }^{i}={𝓍}_{\nabla }^{ini}$ 3: $E_{max}^{o}=\infty$;   // Initialize optimized maximum energy consumption value 4: **For**
${𝓍}_{\nabla }^{i}<\frac{r-\varrho }{2}$
**Do** 5: Calculate energy consumption $E_{d}^{r}=E_{d}^{r}$ of this node using Equation (34);     // Calculate energy consumption $E_{d}^{r}$ at different distance from the sink with the current  r 6: $E_{max}^{\mathrm{cur}}=\max\left(E_{d}^{r}\right)$ //The maximum energy consumption of the current optimal transmission radius set 7: ${𝓍}_{\nabla }^{i}={𝓍}_{\nabla }^{i}-i\times \Delta_{x}$; 8: j=1; 9: **For**
r>ϱ&&r<R
**Do** 10: $r_{new}=r+j\times {𝓍}_{\nabla }^{i}$ 11: **For**
$d=d_{min}$ to R
**Do** //Calculate energy consumption under the current transmission radius $r_{new}$ 12: Calculate energy consumption $E_{d}^{r_{new}}$ of this node using Equation (34) 13: $E_{d}^{r}=E_{d}^{r}+E_{d}^{r_{new}}$; 14: $\bar{E_{d}^{r}}$ = $E_{d}^{r}/j$;    //The average energy consumption after j rotations 15: **End for** 16: $E_{max}^{i}$ = $max\left(\bar{E_{d}^{r}}\right)$      //$E_{max}^{i}$ indicates the maximum energy consumption of $i^{th}$ transmission radius set 17: **IF**
$E_{max}^{i}$ < $E_{max}^{\mathrm{cur}}$ Then  //Maximum energy consumption can be reduced after adding $r_{new}$ 18: $E_{max}^{\mathrm{cur}}=E_{max}^{i}$ 19: **Else** 20: Exit //Stop the search of the current transmission radius set 21: **End if** 22: j=j+1; 23: **End For** 24: **If**
$E_{max}^{o}>E_{max}^{\mathrm{cur}}$ Then    //If the maximum energy consumption of the current transmission set is lower, then record it 25:  $\Upsilon_{o}=\left\{r+{𝓍}_{\nabla }^{o},r-{𝓍}_{\nabla }^{o},\;\;r+2{𝓍}_{\nabla }^{o},r-2{𝓍}_{\nabla }^{o},\;\ldots ,r+j{𝓍}_{\nabla }^{o},r-j{𝓍}_{\nabla }^{o}\;\right\}$ 26: **End if** 27: i=i+1; 28: **End for** 29: **OUTPUT**
$\Upsilon_{o}$, $E_{max}^{o}$

**Figure 14:**
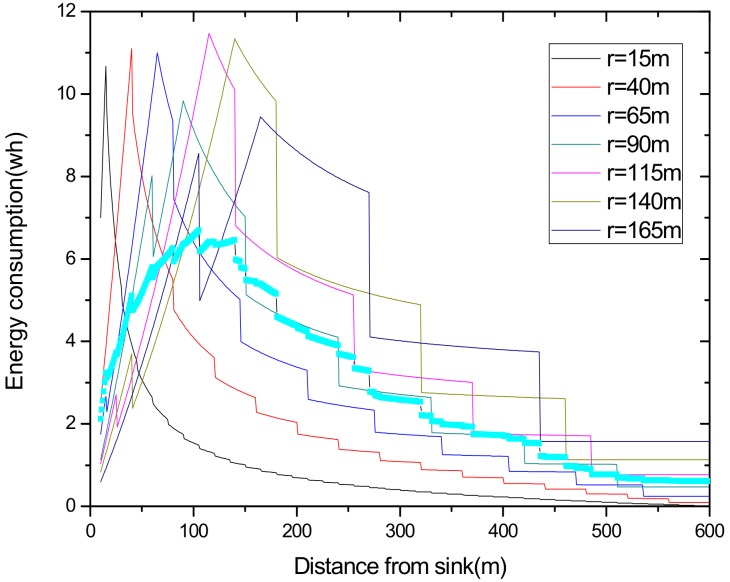
Energy consumption of Algorithm 1.

Using this algorithm, we put an initial transmission radius for the initial r = 90 and ζ=1, getting the radius set {15, 40, 65, 90, 115, 140, 165} in the network with R = 600 m. Figure 14 shows the energy consumption under seven transmission radii, and our adjusted transmission radius according to Algorithm 1. The largest energy consumption achieves 10.6730 wh, 11.1069 wh, 10.9984 wh, 9.8400 wh, 11.4757 wh, 11.3383 wh, and 9.4495 wh of the seven transmission radii. The energy consumption calculated with our algorithm is only 6.69413 wh. It proves that our algorithm effectively reduces the maximum energy consumption of the nodes.

**Figure 15:**
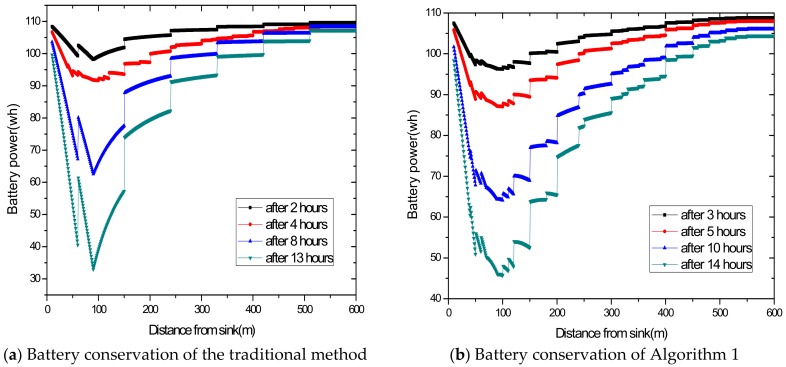
Comparison of battery conservation.

Figure 15 shows that the energy conserving in Algorithm 1 is better than that in the traditional method. For the same battery volume, the residual energy is 92 wh in the traditional method after 2 h, while Algorithm 1 can last for 3 h. And the residual energy is 63.5 wh in the traditional method after 8 h, while Algorithm 1 can last for around 10 h. Since the maximum energy consumption is reduced in Algorithm 1, the energy consumption is obviously less than the traditional method. Thus, for the same battery volume, Algorithm 1 undoubtedly improves the conserving capacity of the battery.

#### 4.2.3. Algorithm Design of Using a Large Transmission Radius to Fully Utilize Energy and Reduce Delay

Algorithm 1 focuses on reducing maximum energy consumption as much as possible. As we all know, it is not only reducing energy consumption that is important, but also utilizing absorbed energy effectively is pivotal in solar-powered wireless sensor networks. Combining the two ideas, we propose energy conserving and transmission radius adaptive protocol (ECTRA) in this section. The scheme is designed to decrease the largest energy consumption at night, and increase energy consumption under the premise that the battery is fully charged during the daytime, by adjusting transmission radius adaptively, according to the available energy at different times.

The way to reduce maximum energy consumption has been discussed in detail; next, we will discuss how to make full use of absorbed energy to further reduce delay. The idea is that when the absorbed energy is predicted to remain, even if the battery has fully charged, a bigger transmission radius, whose maximum energy consumption is less than the available energy, should be adopted to use more energy.

The idea of Algorithm 2 is relatively simple. First, the solar energy that the node can absorb is predicted (here, we address the fact that the prediction of solar energy in this paper is not the main research content, and a little inaccurate prediction on solar energy does not affect the algorithm performance in this paper), and we determine the transmission radius to choose based on the available energy which is determined by solar energy and battery power. Second, when it is predicted that the available energy is not sufficient, such as at night, the data is sent by rotating transmission radius of optimal transmission radius 𝓇 calculated by Algorithm 1. When it is predicted that the available energy is sufficient enough, we send data using a bigger transmission radius to achieve the goal in this paper. Finally, we output the maximum energy consumption of optimal transmission set 𝓇 to compare how much our algorithm can reduce costs.

In order to ensure the remaining battery power of the sensor node is higher than the minimum $v_{\nabla }$ at any time, here, we give the calculation of available energy. Assume t (t∈[0,23]) is the hour of the day, ${ℰ}_{initial}$ is the initial battery power of the node, and ℬ is the battery volume of the node. 𝒽 is the highest observed solar radiation time of the day, and n is the time when sun rises.

Then, to ensure not running out of battery volume before the sun rises, the available energy 𝕖 when there is no more solar energy is

(30) $$𝕖=\frac{{ℰ}_{initial\;}}{n+1}.$$

When the solar radiation is small, such as on a cloudy day, the available energy is

(31) $$A_{t}=\{\begin{array}{c} 𝕖,\;t\in \left[0,𝔫+2\right)\cup \left(𝒽,23\right]\\ {ℱ}^{t},\;t\in \left[𝔫+2,𝒽\right] \end{array}.$$

The available energy in other weather is calculated by

(32) $$A_{t}=\{\begin{array}{c} \;𝕖,\;t\in \left[0,𝔫+2\right)\cup \left(𝒽+2,23\right]\;\\ {ℱ}^{t-1},\;t\in \left[𝔫+2,𝒽\right)\\ {ℱ}^{t-1}\times 0.6,\;t\in \left[𝒽,𝒽+2\right] \end{array}.$$

The specific Algorithm 2 is given below. Here, the meaning of signals used in Algorithm 2 are as follows.

${ℰ}_{max}^{r}$ is the largest energy consumption with the transmission radius r
${𝓇}_{o}$ is a set of optimal transmission radii
${\mathbb{N}}_{d}$ represent the node whose distance from the sink is d
f is the length of data packet, ζ is the reliability
r is the given transmission radius, *R* is the network radius
${𝓍}_{\nabla }^{o}$ is the optimal variable quantity of the transmission radius that can increase or decrease
$\Delta_{y}$ is the increment of transmission radius
${ℱ}^{t}$ is the observed solar radiation at time t of one day
$A_{t}$ is the available energy at time t of one day

**Algorithm 2.** Energy conserving and transmission radius adaptive protocol.INPUT: ${ℱ}^{t}$,$\;\Delta_{y}$, f, ζ, *R*,r, 𝕖OUTPUT: the new maximum energy consumption $E_{max}^{o}$ 1: Set 𝔨=1; 2: Calculate the available energy at time t of one day $A_{j}$ using Equations (31) and (32); 3: Calculate the optimal transmission radius set 𝓇
$=\left\{r+{𝓍}_{\nabla }^{o},r-{𝓍}_{\nabla }^{o},\;\;r+2{𝓍}_{\nabla }^{o},r-2{𝓍}_{\nabla }^{o},\;\ldots ,r+S_{𝓀}{𝓍}_{\nabla }^{o},r-S_{𝓀}{𝓍}_{\nabla }^{o}\;\right\}$ and its maximum energy consumption $E_{max}^{o}$ using Algorithm 1; 4: Calculate the maximum energy consumption ${ℰ}_{max}^{r}$ using Equation (33); 5: **For**
𝑡=0 to 23
**Do** 6: **If**
$A_{t}==𝕖$
**Then**     // Adjusting the transmission radius with transmission radius set 𝓇 7: **For**
$p=1\;\mathrm{to}\;S_{𝓀}$
**Do** 8: $r_{p}=r_{0}+p\times {𝓍}_{\nabla }^{o}$; 9: **For**
$d>r_{p}\&\&d<R$
**Do** 10: Make ${\mathbb{N}}_{d}$ send data with the transmission radius $r_{q}$; 11: **End for** 12: **End for** 13: **Else**   //Make full use of solar energy and reduce delay 14: **While**
r<R   // Find the longest radius that is less than the available energy 15: $r_{new}=r+\Delta_{y}\times k$; 16: Calculate ${ℰ}_{max}^{r_{new}}$ using Equation (33) 17: **Until**
${ℰ}_{max}^{r_{new}}<A_{j}$ 18: **For**
$d>r_{new}\&\&d<R$
**Do**    //Use a bigger transmission radius to improve energy utilization 19: Make ${\mathbb{N}}_{d}$ send data with the new transmission radius $r_{new}$; 20: **End for** 21: **End if** 22: **End for** 23: **If**
$E_{max}^{o}<{ℰ}_{max}^{r}$ Then 24: Output the new maximum energy consumption $E_{max}^{o}$ 25: **End If** 26: **End**

**Figure 16:**
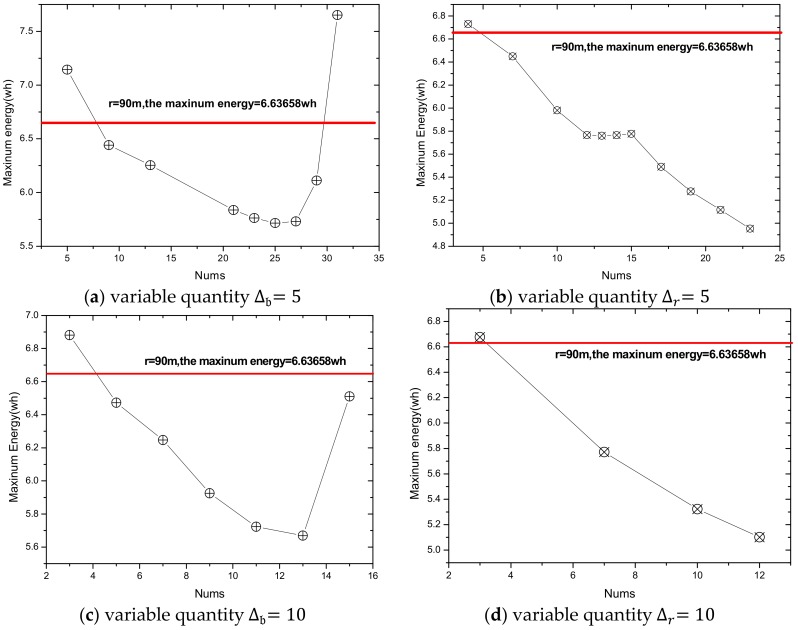

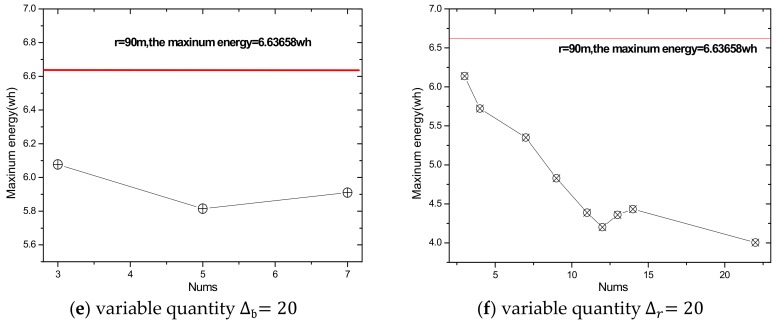
The maximum energy consumption under different variable quantities.

Summarizing the algorithm we proposed, the relevant experimental results are given as follows. In a network with reliability ζ=0.9 and transmission radius r=90 m, we define $\Delta_{b}$ as the variable quantity that increases or decreases, based on the traditional transmission radius and $\Delta_{r}$ as the variable quantity that increases. The maximum energy consumption differs with a different variable quantity. For the same variable quantity, we define nums as the number of added transmission radii. As the number of added radii increases, the maximum energy consumption in the network changes from large to small, and then from small to large. Based on this, we make the Figure 16, in which Figure 16a,c,e indicates the relationship between the number of radii and the maximum energy consumption in the network at night obtained by Algorithm 1, and Figure 16b,d,f indicates the change in maximum energy consumption in the network by only increasing transmission radius during the day.

As can be seen from the figure, at the same variable quantity, we can get a radius number corresponding to the smallest maximum energy consumption, and it varies with a different variable quantity $\Delta_{b}$. When the variable quantity $\Delta_{b\;}=$ 5 or 10, the largest energy consumption with adjusting the radius is bigger than that with the fixed radius. If we stop searching when maximum energy consumption is beyond that with the original radius, we will not go behind, even though there is still a smaller maximum energy behind. When the variable quantity $\Delta_{b}=$ 20, we can get the minimum largest energy consumption of 5.8625 wh. Compared with the minimum values of the maximum energy consumption with $\Delta_{b}$ = 5 or 10, which are 5.771 wh and 5.66921 wh, respectively, it is a little bigger. Therefore, we take full advantage of the law of energy consumption changes, change the condition, and stop searching at the turning point. In this way, we get the optimal transmission radius, set when its maximum energy consumption is 5.66921 wh using our algorithm.

In terms of increasing the transmission radius only, in the case of low reliability, a large amount of data needs to be retransmitted in the near-sink area with a small transmission radius, so the energy consumption is high, which means that increasing the radius reduces the maximum energy consumption. However, as for the network with high reliability, energy consumption may increase sharply as the transmission radius increases, thus, increasing the transmission radius reduces the maximum energy consumption. Our goal during the day is to increase the transmission radius without exceeding the available energy and reduce the delay. Moreover, in actual situations, it is often the case that a large transmission radius is maintained at a certain time to transmit data.

## 5. Performance Analysis and Comparison

### 5.1. Theorem Analysis

Same as the short board effect, which implies that how much water a bucket can hold depends on its shortest board, in the WSN, how long the network lifetime is depends on the max energy consumption. Therefore, finding the node that consumes the most energy makes sense to define the network lifetime.

**Theorem** **3.** *Considering the energy consumption formula we put forward just now, the maximum energy consumption for a transmission radius* r *can be calculated as follows:*

(33) $$E_{max\;}=\tau \left(10𝓈log_{10}\left(\frac{r}{d_{0}}\right)+\mu \right)\left(1+z+\sum_{i=1}^{z}\frac{i}{\zeta^{i}}\right),$$

*where* $\mu =L_{d_{0}}+X_{\sigma }+𝓅-2ln\left[2\left(1-\zeta^{\frac{1}{8f}}\right)\right]\;\times \frac{𝓋}{𝒷}$ *and* $\tau =f\cdot \beta ∕R_{D}$ *in the above formula.*

**Proof.**  According to what we put forward above, the energy consumption calculation formula is summarized as follows:

(34) $$\;E^{total}=\;E^{s}+E^{R}\;\mathrm{where}\{\begin{array}{c} E^{s}=P_{T}\cdot f\cdot D^{S}/𝓋,\\ E^{R}=P_{R}\cdot f\cdot D^{R}/𝓋\\ P_{T}=10𝓈log_{10}\left(\frac{d}{d_{0}}\right)+L_{d_{0}}+X_{\sigma }+𝓅-2\;ln\left[2\left(1-\zeta^{\frac{1}{8f}}\right)\right]\times \frac{𝓋}{𝒷}\\ P_{R}\left(d\right)=P_{T}-10𝓈log_{10}\left(\frac{r}{d_{0}}\right)-L_{d_{0}}+X_{\sigma }\\ D^{S}=\left(1+z+\frac{r}{d}\sum_{i=1}^{z}\frac{i}{\zeta^{i}}\right)\beta ,\;z=\lfloor \frac{R-d}{r}\rfloor \end{array}\;$$

☐

Since we only explore the relationship between the distance from the sink and energy consumption of a node, in order to simply calculate, we can ignore some unnecessary parameters, like data packet f, and replace them with coefficient τ,μ. Through an integrating formula, we can get the following expression:

(35) $$\;E^{total\;}=\{\begin{array}{c} \tau \left(10𝓈log_{10}\left(\frac{d}{d_{0}}\right)+\mu \right)\left(1+z+\frac{r}{l}\overset{z}{\underset{i=1}{\sum }}\frac{i}{\zeta^{i}}\right),\;d<r\\ \tau \left(10𝓈log_{10}\left(\frac{r}{d_{0}}\right)+\mu \right)\left(1+z+\frac{r}{l}\overset{z}{\underset{i=1}{\sum }}\frac{i}{\zeta^{i}}\right),\;d\geq r \end{array}$$

Through the previous calculation, we know that when d is more than r, the transmission power is stable, and data volume decreases as the distance increases. Hence, in the nodes whose distance from the sink is more than r, the node at the distance of r has the largest energy consumption.

As for the nodes whose distance is less than r, we get the maximum point by deriving the function located at the distance of r.

In summary, in the network with a transmission of r, the largest energy consumption appears at the distance r from sink. The following equation is given:

$$\;E_{max\;}=\tau \left(10𝓈log_{10}\left(\frac{r}{d_{0}}\right)+\mu \right)\left(1+z+\overset{z}{\underset{i=1}{\sum }}\frac{i}{\zeta^{i}}\right)$$

**Figure 17:**
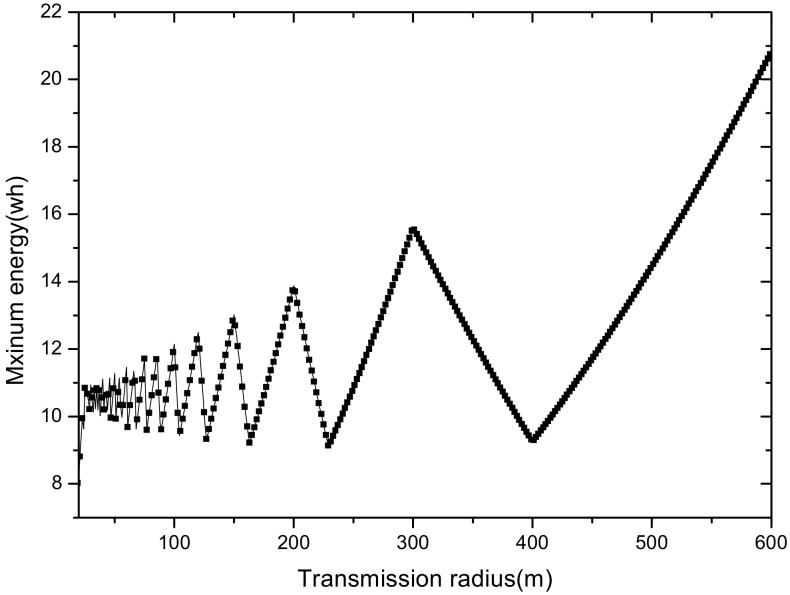
The maximum energy consumption under reliability 1.

Figure 17 shows the trends of maximum energy consumption under different transmission radii with the reliability of 1 when the network radius is 600 m.

**Theorem** **4.** *The total energy consumption with a network radius* R *can be calculated as the following equation:*

(36) $$\begin{array}{ll} E_{sum\;} & =\pi R^{2}\rho \{\overset{r}{\underset{d=1}{\sum }}\left[\epsilon \left(10nlog_{10}\left(\frac{d}{d_{0}}\right)+\mu \right)\left(1+z+\frac{r}{l}\overset{z}{\underset{i=1}{\sum }}\frac{i}{\zeta^{i}}\right)\right]\\ & +\overset{R}{\underset{d=r+1}{\sum }}\left[\;\tau \left(10nlog_{10}\left(\frac{r}{d_{0}}\right)+\mu \right)\left(1+z+\frac{r}{l}\overset{z}{\underset{i=1}{\sum }}\frac{i}{\zeta^{i}}\right)\right]\} \end{array}$$

**Proof.**  To discuss the total energy consumption in the whole network, firstly consider the distribution of individual points on the line l at intervals of 1 m. When the network radius is *R* and the transmission radius is r, the energy consumption on the line is:

(37) $$\;\begin{array}{ll} E_{l} & =\overset{r}{\underset{d=1\;}{\sum }}\left[\tau \left(10𝓈log_{10}\left(\frac{d}{d_{0}}\right)+\mu \right)\left(1+z+\frac{r}{l}\overset{z}{\underset{i=1}{\sum }}\frac{i}{\zeta^{i}}\right)\right]\\ & \;\;\;\;\;\;\;\;\;\;\;\;\;\;\;\;\;\;\;\;\;\;+\overset{R}{\underset{d=r+1}{\sum }}\left[\tau \left(10𝓈log_{10}\left(\frac{r}{d_{0}}\right)+\mu \right)\left(1+z+\frac{r}{l}\overset{z}{\underset{i=1}{\sum }}\frac{i}{\zeta^{i}}\right)\right] \end{array}$$

☐

Assuming that the nodes in the shaded area are distributed with probability ρ, and the network area is $\pi R^{2}$, then the total energy consumption $E_{sum}$ in the whole network is calculated as follows:

$$\begin{array}{c} \;E_{sum}=\pi R^{2}\rho E_{l}\\ \;=\pi R^{2}\rho \{\overset{r}{\underset{d=1\;}{\sum }}[\;\tau \left(10𝓈log_{10}\left(\frac{d}{d_{0}}\right)+\mu \right)\left(1+z+\frac{r}{l}\overset{z}{\underset{i=1}{\sum }}\frac{i}{\zeta^{i}}\right)]\\ \;\;\;\;\;\;\;\;\;\;\;\;\;\;\;\;\;\;\;\;\;\;\;\;\;\;\;\;\;\;\;\;\;\;\;\;\;\;\;\;\;\;\;\;\;\;\;\;\;\;\;\;+\overset{R}{\underset{d=r+1}{\sum }}\left[\;\tau \left(10𝓈log_{10}\left(\frac{r}{d_{0}}\right)+\mu \right)\left(1+z+\frac{r}{l}\overset{z}{\underset{i=1}{\sum }}\frac{i}{\zeta^{i}}\right)\right]\} \end{array}$$

**Figure 18:**
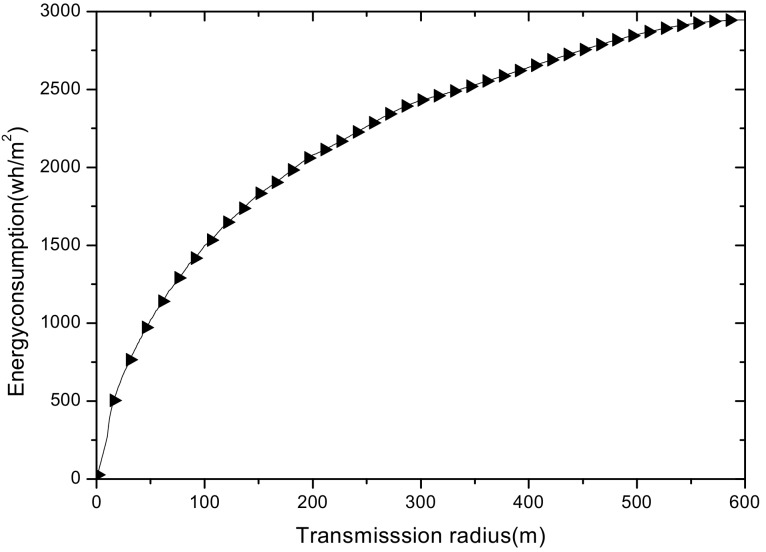
The total energy consumption.

Here, we only calculate the total energy consumption on a straight line to observe the law. As shown in Figure 18, the total network energy consumption increases as the transmission radius increases. It is reasonable because as the transmission radius increases, the required transmission power increases. Although the amount of data decreases, the amount of data reduction is far less than the increase in transmission power when the transmission radius is large.

ECTRA aims to improve the overall energy efficiency, so making full use of the key point that the higher the total energy consumption is, the more suitable the transmission radius is to achieve the goal, is an effective solution. The increase of transmission radius during the day corresponds to this.

### 5.2. Experimental Environment Settings

The following is a comprehensive comparison and analysis of the performance of ECTRA and previous strategies, including energy consumption and utilization comparison, battery power analysis, delay comparison, and cost analysis.

Firstly, we select the solar energy receiver in dimensions of 10 cm × 20 cm. Secondly, we select the day with least solar radiation in recent years as our research subject, which can be obtained in detail from Figure 6. Lastly, we choose a battery that is suitable for each network radius in the following experiment, as listed in Table 1.

### 5.3. Detailed Experiment of ECTRA Scheme

This section gives detailed experimental results of the ECTRA scheme proposed in this paper. Firstly, the experimental environment is reliability ζ=0.9, network radius R=600 m, and transmission radius in traditional method r=90 m. Secondly, in our algorithm, we dynamically adjust the transmission radius to reduce the maximum energy consumption as much as possible during the night, and ensure that the maximum energy is less than available during the day. That is to say, in the case of a battery guaranteed to be fully charged, an increase in transmission radius, and even make the bigger transmission radius fixed at that value. Thus, the network delay is reduced. Next, take the adjustment at night when the available energy is small as an example, and we make the following experiment according to Algorithm 1.

We define Δ as the increasing range of the transmission radius, that is increased to or decreased by the transmission radius in traditional method, and define num as the total number that we have added to our transmission set. Then, the energy consumption is shown, as follows.

**Figure 19:**
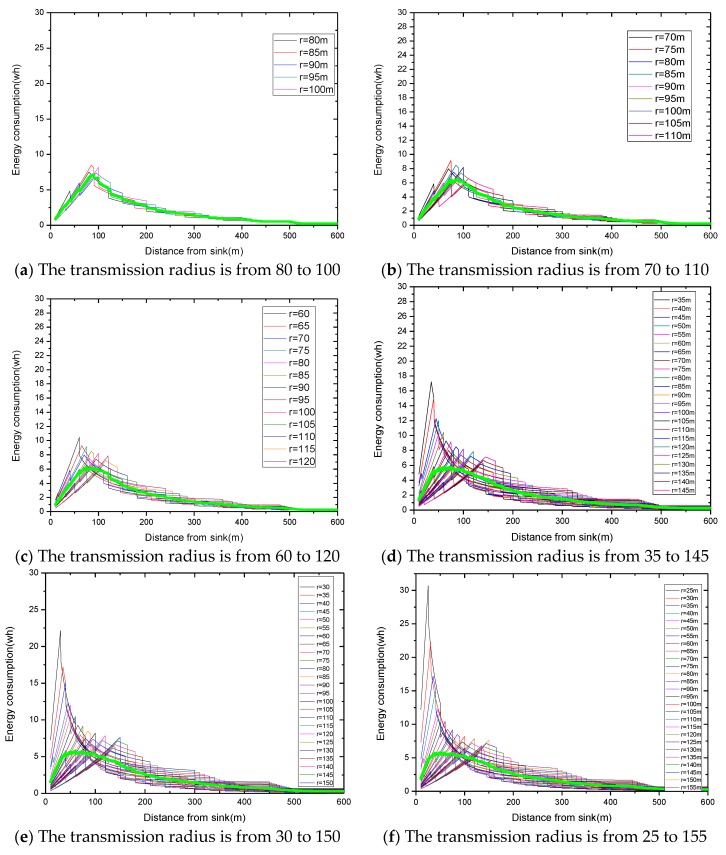
Energy consumption under Δ =5.

**Figure 20:**
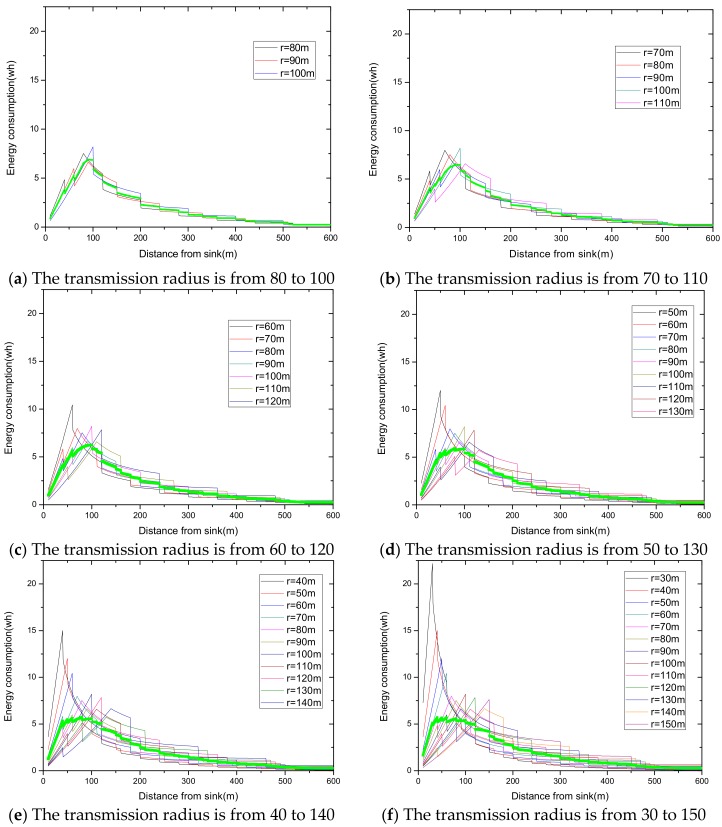
Energy consumption under Δ =10.

**Figure 21:**
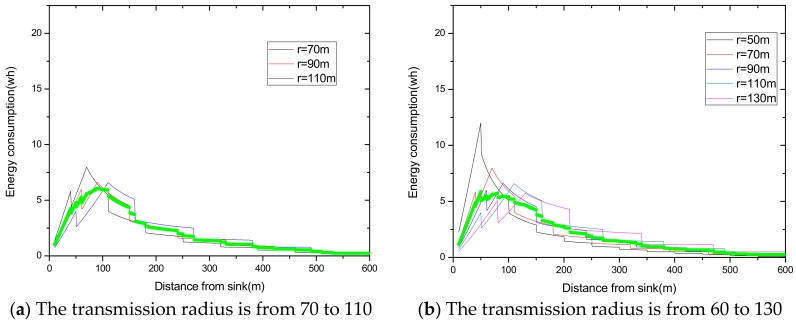

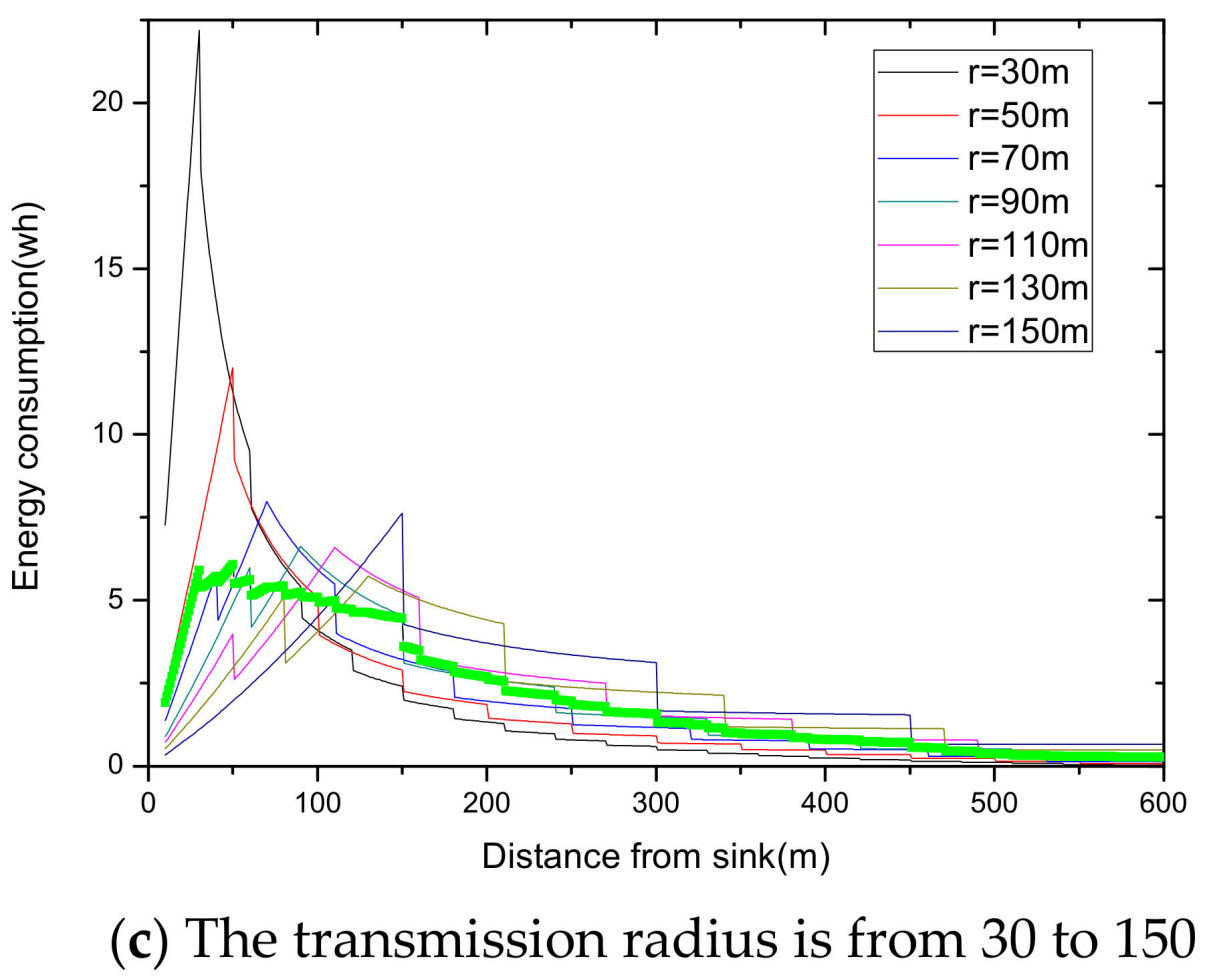
Energy consumption under Δ =20.

Figure 19, Figure 20 and Figure 21 show the energy consumption after we adjust the transmission radius, which is drawn in green. As can be seen from the figure, the energy consumption of each node in our algorithm is lower than the maximum energy consumption of the node in fixed transmission radius, and in general, the maximum energy consumption in our algorithm is lower than the smallest maximum energy consumption of the fixed transmission radius except for a too small or big number. According to Equation (33), the maximum energy consumption with a transmission radius of 90 m is 6.63658 wh. When the increasing range is 10, the maximum energy consumption is 6.88186 wh, 6.47258 wh, 6.2474 wh, 5.92515 wh, and 5.66921 wh in Figure 20a–f, respectively. When the increasing range is 20, the maximum energy is 6.07677 wh, 5.815 wh, and 6.08124 wh in Figure 20a–c respectively. The data shows that the maximum of our adjusted transmission radius can be smaller than the fixed transmission radius of 90 m. Furthermore, the maximum energy consumption is different due to the different numbers of transmission radius and increasing range. Therefore, it is important to find the smallest value in the maximum energy consumption, to decrease the energy consumption as much as possible, which our ECTRA can manage.

Simply considering increasing the transmission radius, the energy consumption under different increasing ranges is shown as follows.

**Figure 22:**
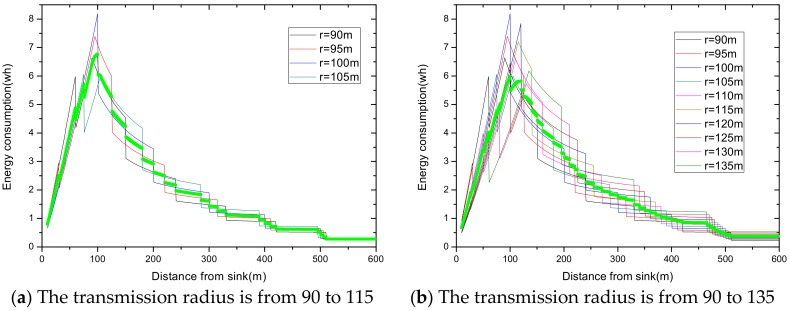

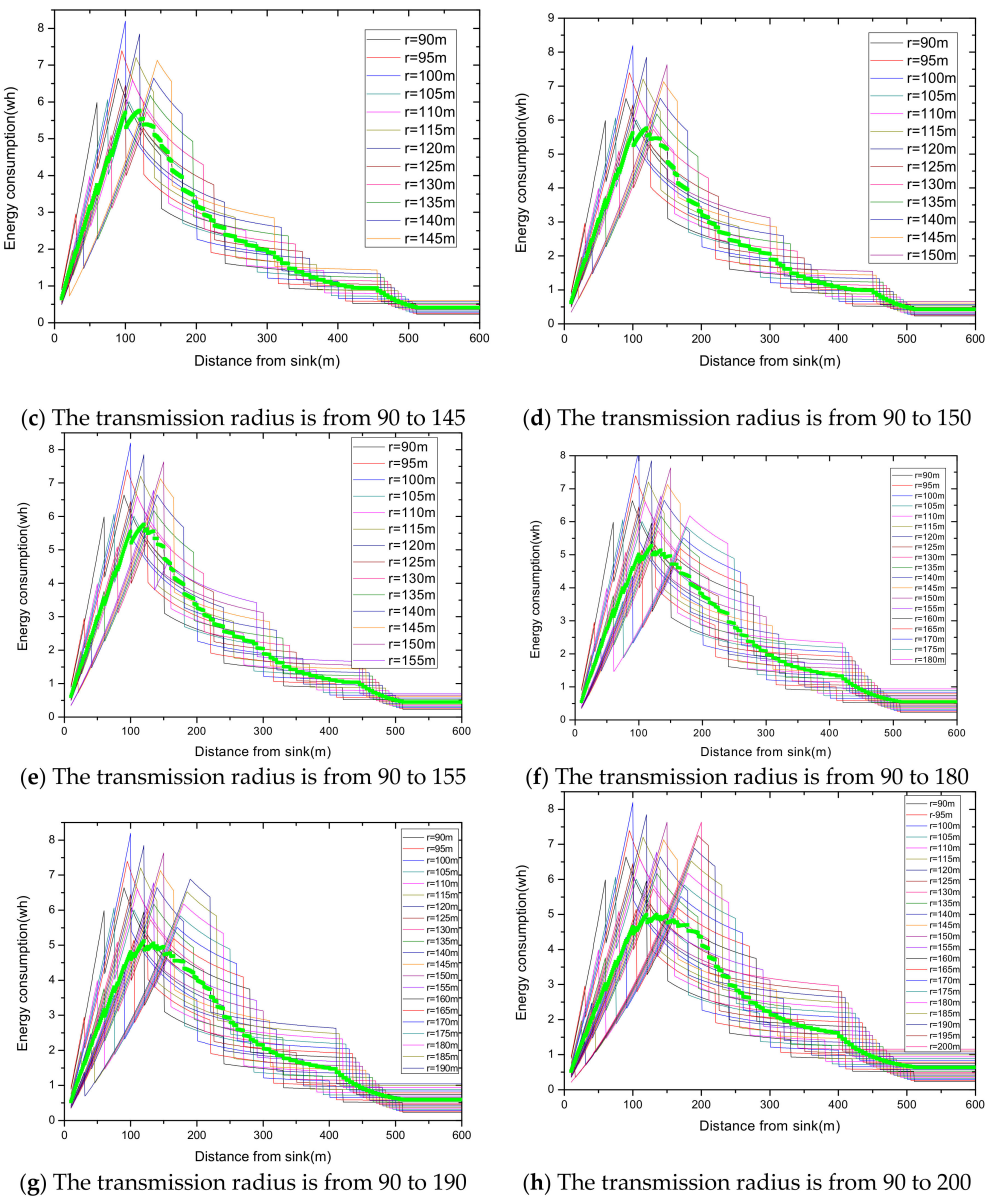
Energy consumption under Δ =5.

**Figure 23:**
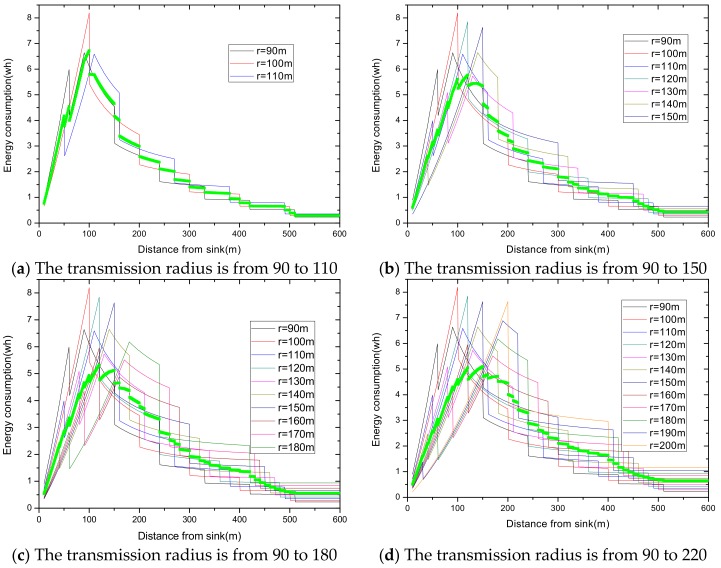
Energy consumption under Δ =10.

**Figure 24:**
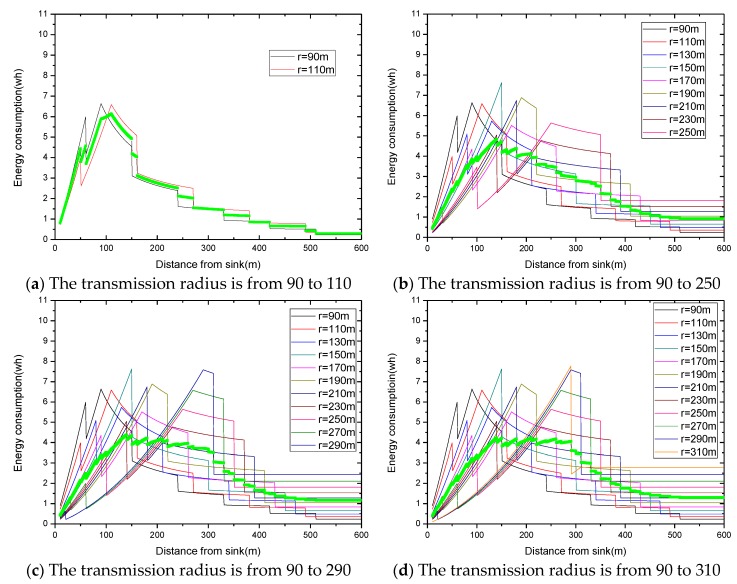

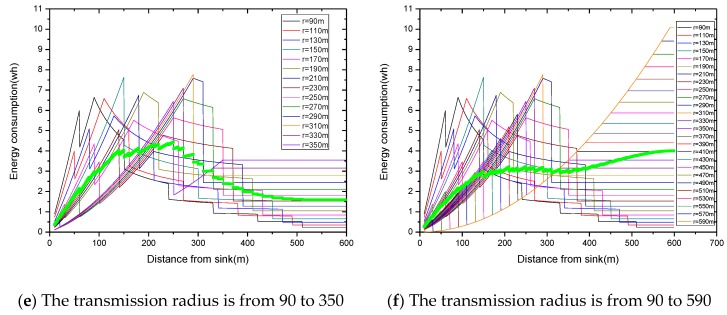
Energy consumption under Δ =20.

When the increasing range is 5, the maximum energy consumption is 6.73 wh, 5.98 wh, 5.76591 wh, 5.76976 wh, 5.76406 wh, 5.2766 wh, 5.11579 wh, and 4.95377 wh, respectively, as shown in Figure 22a–h. When we only add bigger transmission radii, the maximum energy will gradually decrease. Although there may be a turning point in the middle process, the gap is too small to be ignored. However, this is only a case where a large amount of retransmission is required with a low reliability. If the reliability is high, as we add bigger transmission radii, the maximum energy consumption will increase. Specifically, as shown in Figure 23, when the increasing range is 10, the maximum energy consumption is 6.67698 wh, 5.77162 wh, 5.32201 wh, and 5.10115 wh from Figure 23a–d, respectively. When the increasing range is 20, the maximum energy consumption is 6.13934 wh, 4.82823 wh, 4.38661 wh, 4.20 wh, 4.43324 wh, and 4.07641 wh from Figure 24a–f respectively. As we can see, the number of added radii num=12 in Figure 24d is a turning point, whose maximum energy consumption is smaller than that with the number of added radii num=11, and bigger than that with the number of added radii num=13. However, as the number of added radii increases, the maximum energy consumption continues to reduce again, because, as the transmission radius becomes big, the distance of nodes with maximum energy consumption becomes farther and farther from the sink. Compared with Figure 24e,f, although increasing the transmission radius increases the energy consumption of nodes in the far-sink area, the previous nodes with maximum energy consumption locate at a distance around 90 m from the sink. The data load of nodes in near-sink areas decrease due to an increased transmission radius, thus causing the final maximum energy to reduce.

### 5.4. Comparison of Energy Consumption and Utilization

In the following experiment, the transmission radius in the different network in traditional method is listed as follows.

According to the four network radii in Table 2, we have calculated the maximum energy consumption ${ℰ}_{max}$ and battery power ℬ of each network radius under the reliability of 0.98:

The following experimental results give a comparison of the energy utilization rates of the largest energy-consuming nodes in the network, with different network radii and different reliabilities.

**Figure 25:**
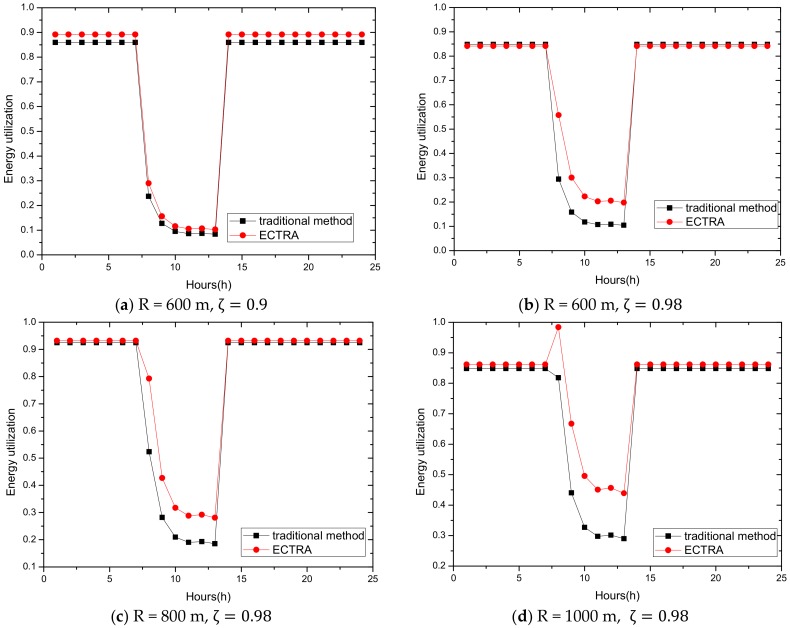
Energy utilization of the nodes with largest energy consumption in a day.

According to Figure 25, in which the energy utilization is calculated by referencing to the data in Table 1 and Table 3, in the whole day, the energy utilization in ECTRA is higher than that in traditional method, no matter how long the network radius is, and how much the reliability is. Since we have changed the battery volume to fit into our new maximum energy consumption at night as calculated by Algorithm 2, the difference from energy utilization between the two methods is small. We increase the transmission radius as long as possible to improve delay without exceeding the available energy, so not only do we decrease the delay, but also improve the energy utilization during the day, as shown in the figure. As the network radius becomes longer, the energy utilization during the day is higher. As the reliability becomes higher, for the same network radius, the energy utilization is higher, because the energy consumption is larger when the reliability is higher. Moreover, we can increase the energy utilization by 55% in the best case. Since the solar energy during the day is too sufficient to make full use of it, and the energy consumption of a node is much smaller compared with the energy that nodes can absorb during the daytime, the energy utilization during the daytime is low. However, according to Theorem 4, the bigger the transmission radius, the higher the total energy consumption. In the case that the solar panel is fixed during the day, the available energy of nodes is the same. That is to say, although the increase in energy utilization on a node is trivial, but in terms of the entire network, it is a large increase in energy utilization.

The following experimental results give a comparison of energy utilization at night for each node.

**Figure 26:**
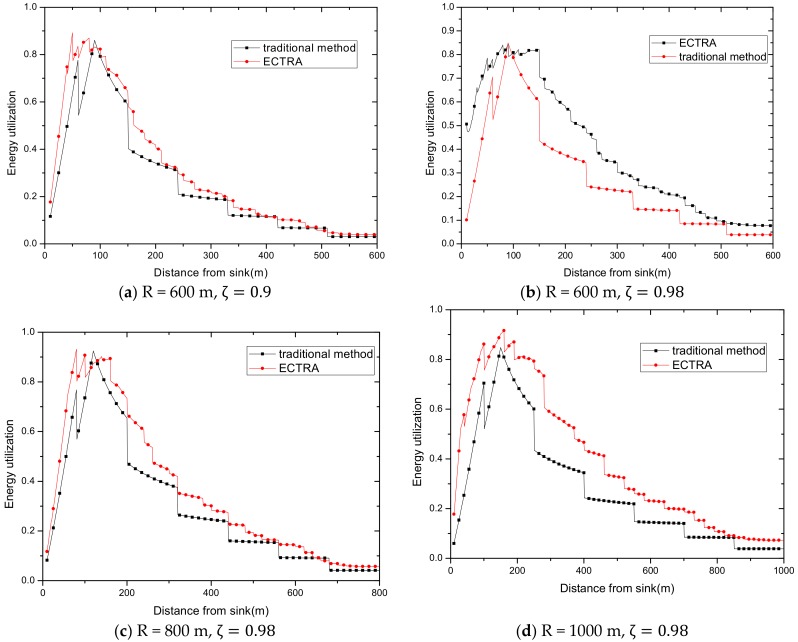
Energy utilization at night.

Obviously, energy utilization appears uneven, as shown in Figure 26. Except for nodes with high energy consumption near the transmission radius, energy utilization of most nodes is below 0.2, which is a tricky problem. Energy utilization of each node is higher in ECTRA than that in the traditional method ranging from 0.05 to 0.2, because we adjusted the transmission radius and calculated the new battery power in time. This relieves the problem of uneven energy utilization to a certain extent. Moreover, the higher the reliability is and the longer the network radius is, the more the energy utilization can be improved. In terms of the whole network, ECTRA can improve energy utilization by 30–60%.

For ordinary panels, because the battery power is relatively small compared to the amount of solar energy, it is always fully charged during the day, and the designers will consider the situation of continuously cloudy days, so that the panel can cope with this bad weather. However, when the maximum energy consumption is large and the required panel power is large, the solar energy provided by the cloudy days cannot make the battery fully charged, resulting in insufficient power not able to support nodes, with outage until the next day. The superiority of our scheme is reflected at this time. Since our scheme achieves a reduction in maximum energy consumption, which can reduce the required battery power, the battery can still be fully charged in the case of continuously cloudy days. Taking the node with the largest energy consumption as the research goal, we give the research results of battery power in the traditional method and ECTRA scheme.

**Figure 27:**
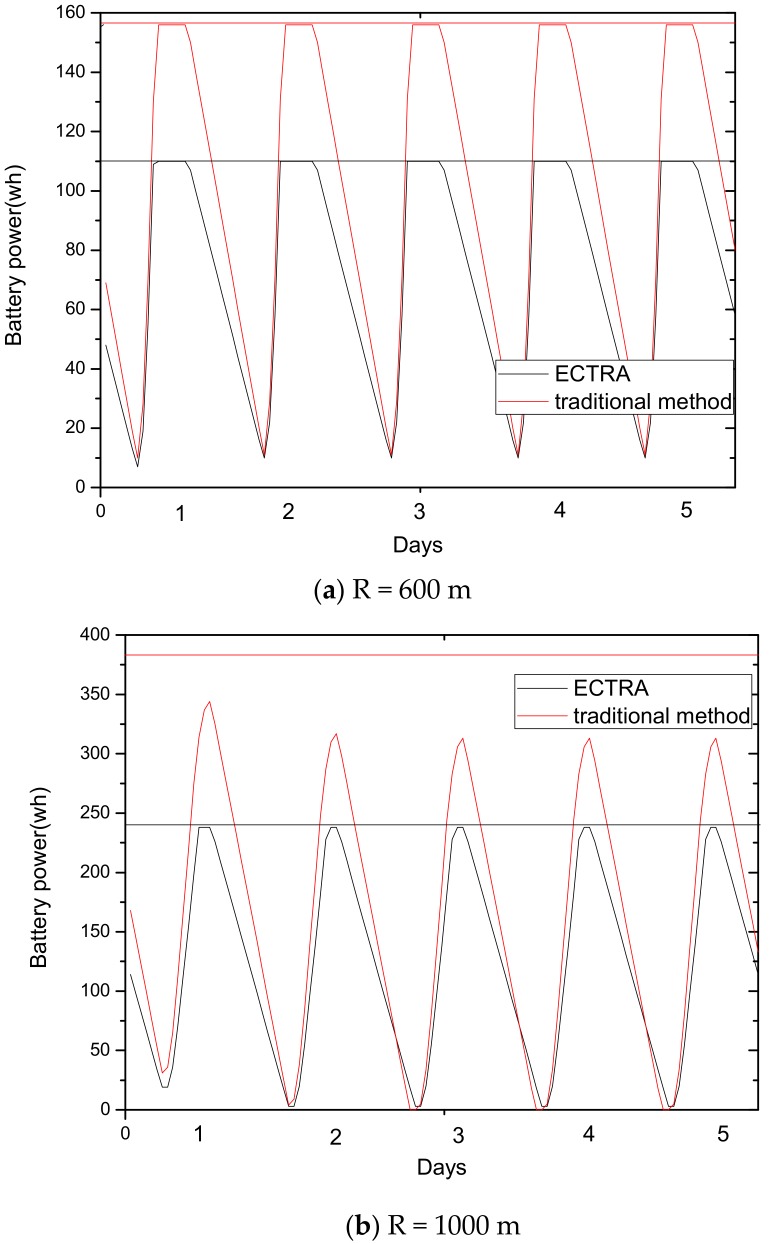
Battery power for five consecutive cloudy days.

The battery volume is listed in Table 1. Assume that the initial battery power is 191 wh in the traditional method, and 79 wh in ECTRA when the network radius is 1000 m; and 131 wh in traditional method, and 51 wh in ECTRA when the network radius is 600 m. As shown in Figure 27, In the case of five continuously cloudy days, the battery electricity in both methods with a network radius of 600 m can be fully charged, and does not run out. However, the gap appears when the network radius is 1000 m. As can be seen from Figure 27b, in the traditional scheme, the battery power can only last for one day, and runs out at 6:00 the next day. Since the battery cannot be fully charged during the daytime, it runs out at 5:00 and 6:00 in the following four days. Not only does the battery in our ECTRA scheme not have a power outage, but it is also fully charged at noon on the 2nd to 5th day. This fully demonstrates the effectiveness of ECTRA, which reduces the power consumption by 37.36%, and also remains stable on continuously cloudy days.

### 5.5. Comparison of Network Delays

The comparison of the delays under different strategies is given in this section.

**Figure 28:**
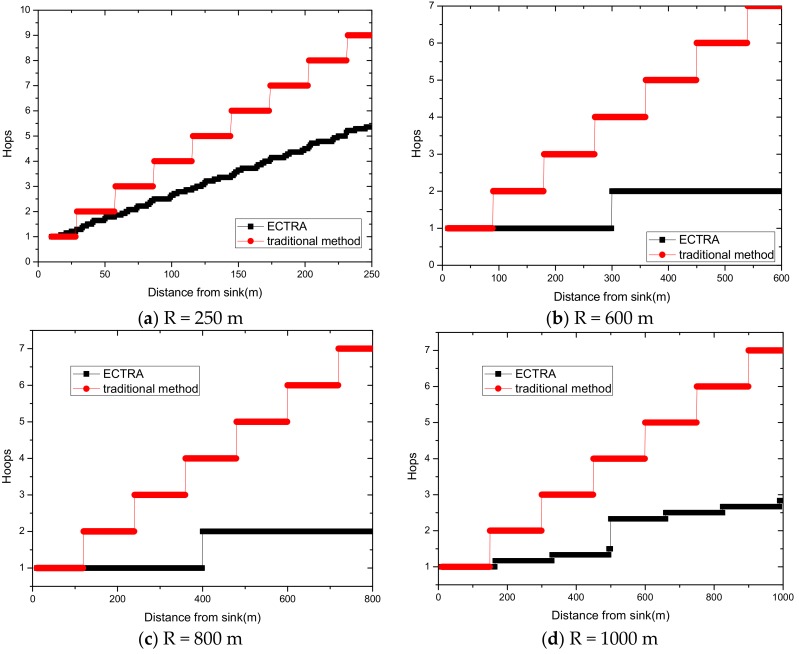
Network delays.

As shown in Figure 28, the network delay is smaller in ECTRA than that in the traditional method. Since we reduce the delay by increasing the transmission radius directly during the day, and maintain the delay in balance at night by increasing and reducing the radius, the delay in a whole day is reduced. In general, ECTRA decreases the delays by 44% to 71%. In order to decrease delays and ensure the battery is fully charged, in a 250 m network, we send data directly at a transmission radius of 250 m. In the networks with a radius of 600 m and 80 m, we send data with a transmission radius of 300 m and 400 m, respectively. In the network of 1000 m, the transmission radius of 165 m is chosen to transmit data when sun rises, and in the next few hours during the day, the available energy is sufficient to support energy consumption of a transmission radius of 500 m.

### 5.6. Comparison of Costs of the Battery

In a wireless sensor network, the battery’s power determines the how much the largest energy consumption can be in the entire network. However, this does not mean that we can constantly increase the amount of electricity in order to have the largest energy consumption, because battery power is also subject to cost constraints. The higher the battery’s power, the higher the cost of the battery. Here, we use a scale factor ϑ to define the relationship between the battery power and the cost, and we assume ϑ is valued at 0.002. If we define the battery cost for a node with a battery volume of 10 wh is $C_{0}$, which is assumed as 0.2 dollar here, then we can use the following formula to express the relationship

(38) $$C=C_{0}+\vartheta \times \frac{ℬ}{{ℬ}_{0}},$$

where ${ℬ}_{0}$ is the reference battery volume, and ℬ is the minimum battery volume of a sensor node. It can be seen from the above formula that if the battery volume is reduced, the cost can be reduced to some extent. Also, our protocol can reduce the maximum energy consumption and further reduce costs.

According to this equation, we calculate the cost of a battery in the four network radii and compare the difference between traditional method and ECTRA to see how much our scheme can save in costs.

**Figure 29:**
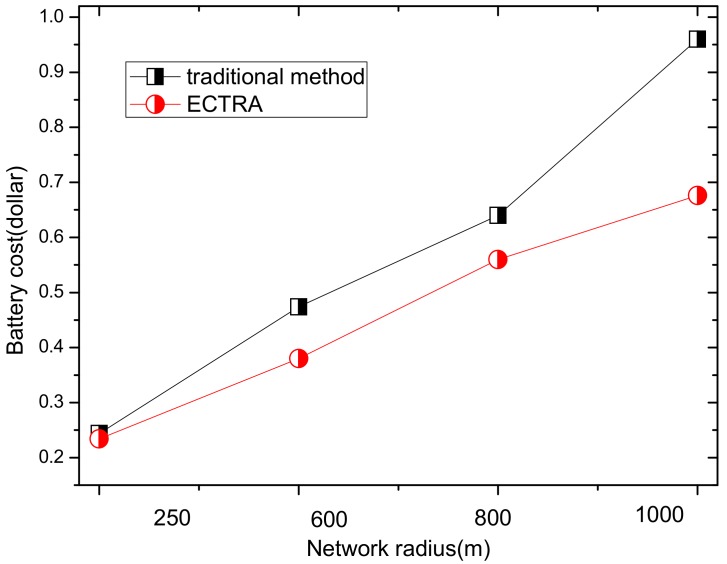
The battery costs on a sensor node under four network radii.

As shown in Figure 29, the battery cost on a sensor node in ECTRA is less than that in the traditional method. As the network radius is larger, the cost is much lower. When the network radius is 1000 m, the battery cost in the traditional method is 0.96 dollars, while the battery cost in ECTRA is 0.676 dollars, reduced by 29.58% and saving 0.284 dollars on a sensor node. Although it may seem little, in the EHWSNs, where a large number of sensor nodes are deployed in the network, a lot of capital investment in battery cost can be saved.

## 6. Conclusions

With the development of microelectronic technology, the internet of things (IoT) based on sensors is becoming a pervasive paradigm. Energy is always a hot study issue in a sensor-based network. Although in energy harvesting wireless sensor networks, nodes can absorb energy from the surrounding environment and enable sensor-based network to achieve long-term monitoring in unattended environments, it is not an ignorable problem to reduce construction cost on sensor nodes as much as possible, considering economic costs, deployment space, etc. Therefore, energy conservation is still a significant but challenging subject for study. The ECTRA scheme is proposed in this paper, which can effectively reduce the network energy requirements, effectively utilize the energy absorbed from the surrounding environment, and reduce the deployment costs by adjusting the transmission radii of nodes. Moreover, the theoretical analysis confirmed the validity of the ECTRA scheme. Innovation of the ECTRA scheme lies in two points. The first is rotating the nodes of maximum energy consumption in the network by adjusting the transmission radius adaptively to achieve a reduction in the energy consumption of nodes. The second is increasing the transmission radius moderately in the case where the solar energy supply is adequate enough to reduce the energy consumption of nodes in near-sink areas, and reduce network delays by reducing the hops required for the packet to arrive at the sink. In summary, the ECTRA scheme has a good comprehensive performance. However, although improving the network performance by adjusting the transmission radius, which is designed in ECTRA scheme in this paper, is a simple and effective strategy, a better optimal result can be obtained if considering optimization from multiple layers of the network. Therefore, we will conduct further research on how to optimize the performance of EHWSNs from cross-layers.

## Figures and Tables

**Table 1:** Battery power between traditional method and ECTRA.

Network Radius (m)	250	600	800	1000
ℬ in traditional method (wh)	22	137	220	380
ℬ in ECTRA (wh)	17	110	180	238

**Table 2:** The transmission radius of the corresponding network radius in traditional method.

Network Radius (m)	250	600	800	1000
Transmission radius (m)	29	90	120	150

**Table 3:** The maximum energy consumption between the traditional method and energy conserving and transmission radius adaptive (ECTRA) scheme.

Network Radius (m)	250	600	800	1000
${ℰ}_{max}\;\mathrm{in}\;$traditional method (wh)	1.506	8.2474	14.6601	22.9026
${ℰ}_{max}\;$ in ECTRA (wh)	1.134	5.4199	11.9772	16.5128
